# Age‐stratified machine learning identifies divergent prognostic significance of molecular alterations in AML

**DOI:** 10.1002/hem3.70132

**Published:** 2025-05-07

**Authors:** Jan‐Niklas Eckardt, Waldemar Hahn, Rhonda E. Ries, Szymon D. Chrost, Susann Winter, Sebastian Stasik, Christoph Röllig, Uwe Platzbecker, Carsten Müller‐Tidow, Hubert Serve, Claudia D. Baldus, Christoph Schliemann, Kerstin Schäfer‐Eckart, Maher Hanoun, Martin Kaufmann, Andreas Burchert, Johannes Schetelig, Martin Bornhäuser, Markus Wolfien, Soheil Meshinchi, Christian Thiede, Jan Moritz Middeke

**Affiliations:** ^1^ Department of Internal Medicine I University Hospital Carl Gustav Carus, TUD Dresden University of Technology Dresden Germany; ^2^ Else Kröner Fresenius Center for Digital Health TUD Dresden University of Technology Dresden Germany; ^3^ Center for Scalable Data Analytics and Artificial Intelligence (ScaDS.AI) Dresden/Leipzig Dresden Germany; ^4^ Institute for Medical Informatics and Biometry TUD Dresden University of Technology Dresden Germany; ^5^ Translational Science and Therapeutics Division Fred Hutchinson Cancer Research Center Seattle Washington USA; ^6^ Division of Hematology and Oncology Seattle Children's Hospital Seattle Washington USA; ^7^ Department of Hematology, Cellular Therapy, Hemostaseology and Infectious Disease University of Leipzig Medical Center Leipzig Germany; ^8^ Department of Medicine V University Hospital Heidelberg Heidelberg Germany; ^9^ Department of Medicine 2, Hematology and Oncology Goethe University Frankfurt Frankfurt Germany; ^10^ Department of Hematology and Oncology University Hospital Schleswig Holstein Kiel Germany; ^11^ Department of Medicine A University Hospital Münster Münster Germany; ^12^ Department of Internal Medicine V Paracelsus Medizinische Privatuniversität and University Hospital Nürnberg Nürnberg Germany; ^13^ Department of Hematology University Hospital Essen Essen Germany; ^14^ Department of Hematology, Oncology and Palliative Care Robert Bosch Hospital Stuttgart Germany; ^15^ Department of Hematology, Oncology and Immunology Philipps‐University Marburg Marburg Germany; ^16^ German Cancer Consortium (DKTK), Partner Site Dresden German Cancer Research Center (DKFZ) Heidelberg Germany; ^17^ National Center for Tumor Diseases (NCT), NCT/UCC Dresden, a partnership between DKFZ Faculty of Medicine and University Hospital Carl Gustav Carus, TUD Dresden University of Technology, and Helmholtz‐Zentrum Dresden‐Rossendorf (HZDR) Germany

## Abstract

Risk stratification in acute myeloid leukemia (AML) is driven by genetics, yet patient age substantially influences therapeutic decisions. To evaluate how age alters the prognostic impact of genetic mutations, we pooled data from 3062 pediatric and adult AML patients from multiple cohorts. Signaling pathway mutations dominated in younger patients, while mutations in epigenetic regulators, spliceosome genes, and *TP53* alterations became more frequent with increasing age. Machine learning models were trained to identify prognostic variables and predict complete remission and 2‐year overall survival, achieving area‐under‐the‐curve scores of 0.801 and 0.791, respectively. Using Shapley (SHAP) values, we quantified the contribution of each variable to model decisions and traced their impact across six age groups: infants, children, adolescents/young adults, adults, seniors, and elderly. The highest contributions to model decisions among genetic variables were found for alterations of *NPM1*, *CEBPA*, inv(16), and t(8;21) conferring favorable risk and alterations of *TP53, RUNX1, ASXL1*, del(5q), ‐7, and ‐17 conferring adverse risk, while *FLT3*‐ITD had an ambiguous role conferring favorable treatment responses yet poor overall survival. Age significantly modified the prognostic value of genetic alterations, with no single alteration consistently predicting outcomes across all age groups. Specific alterations associated with aging such as *TP53*, *ASXL1*, or del(5q) posed a disproportionately higher risk in younger patients. These results challenge uniform risk stratification models and highlight the need for context‐sensitive AML treatment strategies.

## Graphical abstract

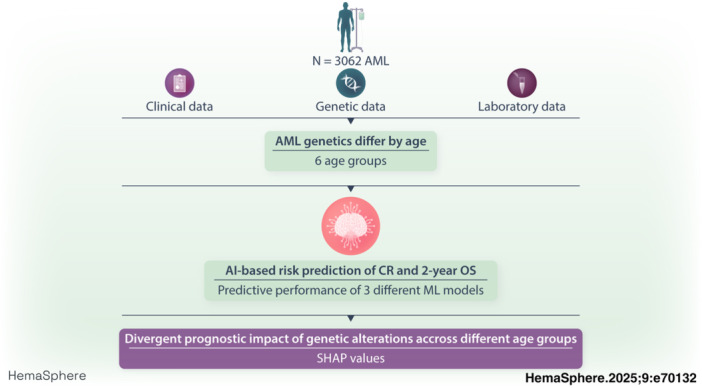

## INTRODUCTION

Adjusted for age, acute myeloid leukemia (AML) affects 4.3 per 100,000 persons in the United States every year.^1^ Despite recent advances in personalized and targeted therapies, patient outcomes still remain largely unsatisfactory.^2^, ^3^ Age plays a major role in both AML biology and patient management. The incidence of AML increases with age, posing a major challenge for healthcare systems in light of the uniformly aging population in industrialized countries.^4^, ^5^ The median age at initial diagnosis is estimated to be 68 years.^6^ In patients over 65 years of age, incidence rates rise to 20.1 per 100,000 persons.^1^ Meanwhile, age remains a major criterion in treatment decisions, as many intensive chemotherapy‐based regimens or allogeneic stem cell transplantation (ASCT) are less tolerable for older patients due to frailty or comorbidities.^7^ A better understanding of disease biology, based on numerous studies of the individual impact of cytogenetic and molecular genetic alterations on prognostication, has led to refined treatment recommendations. Given the advancements in molecular analysis, guidelines such as the European Leukemia Net (ELN) recommendations^8^, ^9^, ^10^ have been continuously improved over the last decade. Nevertheless, following these guidelines often mirrors decision‐making along the nodes of a binary decision tree. First, the absence or presence of a particular mutation is noted. Second, if the mutation is classified within the guidelines as, for example, favorable and other risk‐defining mutations are absent, the patient is assigned to the favorable risk group. Third, this risk stratification is used to inform treatment decisions. While we do not aim to replace validated clinical decision‐making models, we aim to demonstrate their limitations and advocate for more personalized models incorporating multidimensional patient variables in a data‐driven manner.

Given the disparity between tolerability of intensive regimens, these treatment recommendations differ between pediatric and adult patients.^10^, ^11^, ^12^ Nevertheless, the age‐dependent impact of cytogenetic aberrations or molecular alterations is often neglected in risk stratification, despite evident discrepancies in the molecular landscape of pediatric and adult AML.^13^, ^14^ Studies in pediatric AML often fail to compare results to adult AML and vice versa. Studies in adult AML seldom stratify age groups beyond the common threshold of 65 years of age, where patients are deemed less eligible for intensive therapy.^15^ As patient age modifies disease biology, we hypothesize that risk stratification based on cytogenetic and molecular alterations may be context‐sensitive regarding patient age.

## MATERIALS AND METHODS

### Patient data

To identify the age‐dependent impact of genetic alterations, we analyzed a total of 3062 patients with pediatric or adult AML from the German Study Alliance Leukemia (SAL), the Fred Hutchinson Cancer Research Center Seattle, as well as publicly available data from Bottomly et al.^16^ and TARGET data originally published by the National Cancer Institute.

In the first cohort obtained from the German SAL, clinical, laboratory, and genetic data were obtained from 1606 adult patients that were treated within previously conducted multicentric prospective clinical trials (AML96 [NCT00180115],^17^ AML2003 [NCT00180102],^18^ AML60+ [NCT00180167],^19^ and SORAML [NCT00893373]^20^) registered in the SAL registry (NCT03188874). Table S1 shows an overview of trial protocols. In the original clinical trial protocols, only patients ≥18 years of age were eligible. Acute promyelocytic leukemia (APL, syn. AML‐M3) patients were excluded from these trials. All patients gave their written informed consent according to the revised Declaration of Helsinki.^21^ All studies were previously approved by the Institutional Review Board of the TUD Dresden University of Technology (EK 98032010). Complete remission (CR) and overall survival (OS) were defined according to the revised ELN criteria.^10^

In the second cohort obtained from the Fred Hutchinson Cancer Center Seattle, clinical, laboratory, and genetic data were available for 578 pediatric patients from the Children's Oncology Group (COG) protocol, AAML0531 (NCT01407757).^22^ All subject samples were obtained by member COG institutions after written consent was obtained from the parents/guardians of minors upon enrolling in the trial. The genomic study was overseen by the Institutional Review Board at Fred Hutchinson Cancer Research Center (protocol 9365). Data on selected clinical (e.g., age, presenting hematological indices and cytogenetic classification) and molecular (e.g., *KIT*, RAS genes, *NPM1*, *WT1*, *CEBPA* mutations, and *FLT3*‐ITD allelic ratios) features were clinically available before additional genomic analyses were performed.

Both cohorts were pooled with publicly available data from Bottomly et al.^16^ (*n* = 728) and 150 patients from TARGET‐AML, ^23^ both accessed via cbioportal.com in October 2023 and comprising clinical data, as well as whole genome or whole exome sequencing data. Notably, all pediatric patients and adult patients from the SAL cohort were treated with intensive chemotherapy, while 15.9% of patients within the cohort derived from Bottomly et al. were treated with non‐intensive regimens. Baseline demographic and clinical characteristics of the cohorts are displayed in Table S2.

### Molecular analyses

For the SAL cohort, biomaterial was obtained from bone marrow aspirates or peripheral blood before treatment initiation. Next‐generation sequencing (NGS) was conducted using the TruSight Myeloid Sequencing Panel (Illumina, San Diego, CA, USA). A comprehensive list of covered alterations is provided in Table S3. Pooled samples were sequenced paired‐end and a 5% variant allele frequency mutation calling cut‐off was used with human genome build hg19 as a reference, as previously described in detail.^24^ Additionally, high resolution fragment analysis for *FLT3*‐ITD,^25^ *NPM1*,^26^ and *CEBPA* ^27^ was performed as described previously. For cytogenetics, standard techniques for chromosome banding and fluorescence in situ hybridization were used.

For the cohort from the Fred Hutchinson Cancer Center Seattle, targeted capture sequencing was performed for a panel of over 400 cancer‐related genes. Clinical sequencing of prognostically relevant alterations such as *FLT3*‐ITD, *NPM1* 4bp‐insertion, and mutations in *CEBPA* was performed as previously described.^13^

As only NGS panel data were available for SAL patients, we focused on common genes listed in Table S3 and cytogenetics available for all cohorts. Finally, all patients were categorized into six age groups: infants (0–2 years), children (3–14 years), adolescents and young adults (AYA, 15–39 years), adults (40–64 years), seniors (65–74 years), and elderly (75+ years). Age ranges were guided by definitions of the American Society of Pediatrics^28^ and the National Cancer Institute.^29^ The conventional binarization of adult AML patients at 65 years was included, however, given the advances in patient management and supportive therapies, patients beyond 65 years of age may still be eligible for intensive therapies yielding improved outcomes.^30^, ^31^, ^32^ To account for this paradigm shift, an additional binarization was introduced at 75 years. We acknowledge that further categories are conceivable, but additional subdivision leads to smaller group sizes, which destabilize the model training process since there are fewer subjects per group to train on.

### Statistical analysis

All tests were carried out as two‐sided tests. Statistical significance was determined using a significance level *α* of 0.05. Multiple hypothesis testing was adjusted for using the Benjamini–Hochberg method.^33^ Univariate analyses for binary outcomes (CR rate) were carried out via LR to obtain odds ratios (OR) and 95% confidence intervals (95% CI). Time‐to‐event analyses (OS) were carried out using Cox proportional hazard models to obtain hazard ratios (HR) and 95% CI. Statistical analysis was conducted using R version 4.2.3 and RStudio version 2023.12.0‐369.

### Explainable Machine Learning (ML)

Binary classifications were applied for the achievement of CR after intensive induction therapy, as well as for OS binarized at the 2‐year mark (2‐year OS). Patients with missing CR status were excluded for CR prediction (*n* = 191), and patients that were censored before 2 years of follow‐up were excluded for 2‐year OS prediction (*n* = 204). Missing values were imputed using a *k*‐nearest neighbor (KNN) imputer with *k* = 5. The dataset was separated into 80% training and 20% test data, stratified by age groups and the outcome of interest. To reduce model dimensionality, which may otherwise destabilize the training process (see “curse of dimensionality”^34^), features with a frequency of <1% were excluded. ML models used include Random Forest (RF), Logistic Regression (LR), and XGBoost (XGB). LR is a linear model that predicts binary outcomes based on a weighted combination of input variables and assumes a linear relationship between predictors and the log‐odds of the outcome. RF is an ensemble learning method that builds multiple decision trees during training and outputs the mode of classifications or the average of predictions, effectively capturing nonlinear relationships and interactions between variables. XGB is a gradient‐boosted decision tree method that sequentially builds trees to minimize prediction errors, offering high flexibility in handling complex, high‐dimensional data. Hyperparameter optimization was performed for each model using a 10‐fold cross‐validation on the training set. Model performance was measured using the area under the receiver‐operating characteristic (AUROC), as well as the Matthew's correlation coefficient (MCC)^35^ on the test set. For each non‐deterministic model (RF and XGB), we report the average classification performance and the range of 100 runs to provide the most robust result instead of reporting highly‐performant outlier runs that may occur due to random variation in the training process. Explainability (i.e., the causal effect of a feature's contribution to model inference) was quantified using Shapley (SHAP) values. These are derived from game theory, where they provide a measure of how much credit is allocated to each individual model feature at the input level, given a specific output for binary endpoints. Therefore, they allow to measure and rank the importance of each feature, in our case genetic alterations, for model performance. We provide the SHAP values for the model with most average results (according to MCC) of 100 runs to ensure a balanced representation of feature importance. To understand how each feature influenced the ML model's decisions across different age groups, we first calculated the SHAP values for each feature within each age group. Next, we summed the SHAP values for each feature across all age groups. Finally, we expressed the contribution of each age group as a percentage of the total SHAP values for that feature.

## RESULTS

### Altered genetic pathways differ by age groups

**Figure 1:**
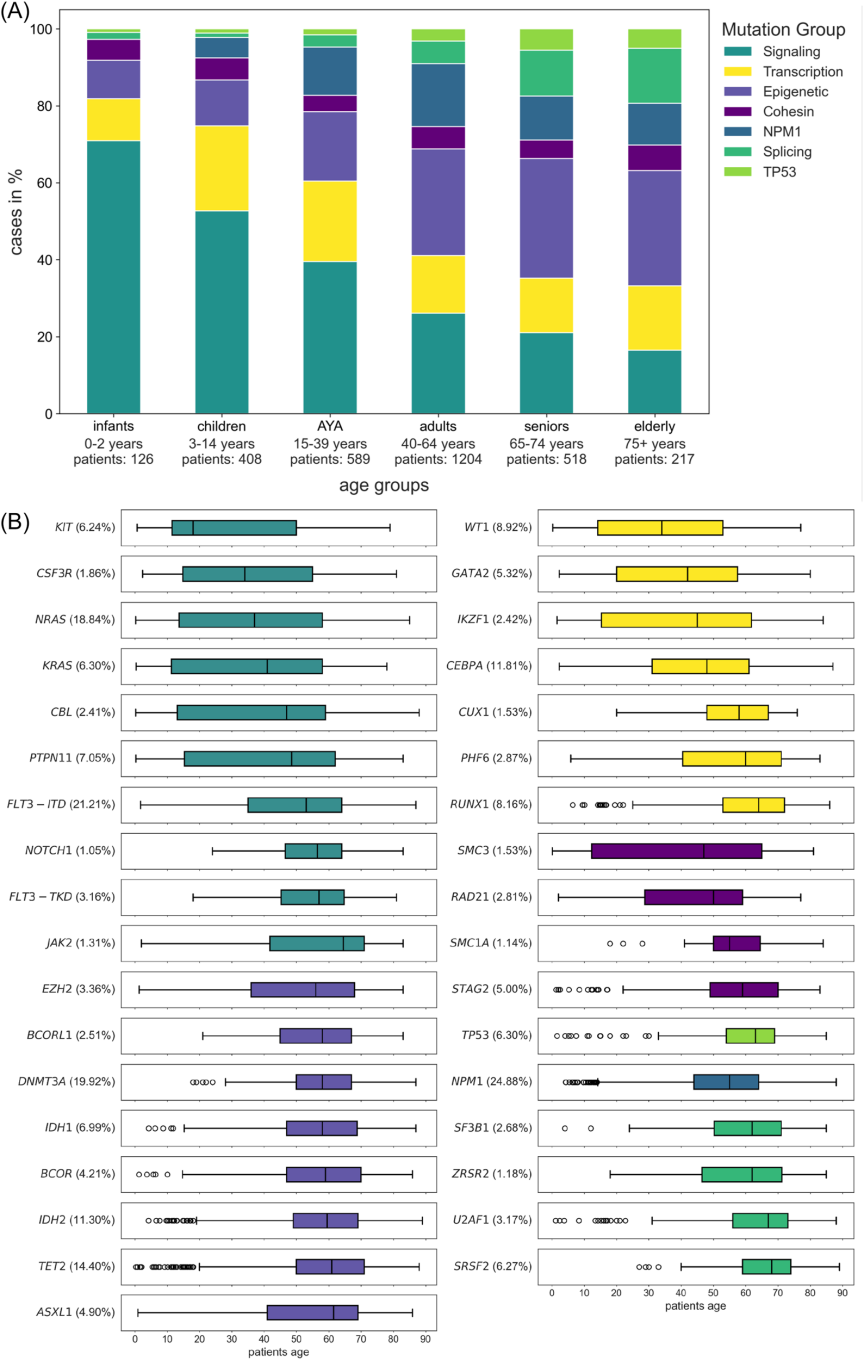
**Distribution of molecular alterations across age groups.** Genetic alterations are grouped according to their function, and their distribution is compared between age groups **(A)**. Alterations of signaling pathways were more prevalent in younger patients and decreased with age, while alterations of epigenetic regulators, the spliceosome, and *TP53* were rare in younger patients and continuously increased with age. Boxplots show the age distribution of each individual alteration **(B)**. Boxplot: bold vertical line, median; box, interquartile range (IQR, i.e., 25th to 75th percentile); lower whisker, Q1 – 1.5 ∗ IQR; upper whisker, Q3 + 1.5 ∗ IQR; dots, outliers.

Among all 3062 patients in our pooled cohort, infants constituted 4.1% (*n* = 126), children 13.2% (*n* = 408), AYA 19.2% (*n* = 589), adults 39.3% (*n* = 1204), seniors 16.9% (*n* = 518), and elderly 7.1% (*n* = 217). For a comprehensive overview of the genetic landscape per age group, molecular alterations were grouped according to the affected cellular functions (Figure 1A) in accordance with previous definitions.^36^ Alterations in signaling pathways were most frequently found in younger patients and their frequency decreased with age. More than half of the pathogenic alterations found in infants (70.9%) and children (52.7%) affected signaling pathways, whereas only 21.1% of seniors and 16.5% of elderly patients carried such alterations. Vice versa, the frequency of alterations affecting epigenetic regulators or the spliceosome, as well as *TP53* alterations increased with age. Alterations in transcription factors were most frequently found in children (22.1%) and AYA (20.9%), while alterations of *NPM1* were most frequent in adults (16.4%) and AYA (12.5%). Alterations in genes of the cohesin complex were equally distributed across age groups. Figure 1B shows the age distribution for individual molecular alterations. The lowest median age was found for *KIT* alterations at 18 years (interquartile range [IQR]: 11–51), while alterations of *SRSF2* had the highest median age at 68 years (IQR: 59–74). The median age for patients with *NPM1* alterations was 55 years (IQR: 44–64), similar to those with *FLT3*‐ITD at 53 years (IQR: 35–64), while patients with *TP53* alterations had a median age of 63 years (IQR: 54–69). Cytogenetics were categorized into ELN2022 risk groups.^10^ The highest frequency of favorable risk cytogenetics was found in children and decreased with age, while adverse risk cytogenetics were frequently found in infants followed by seniors and elderly patients (Figure S1A). The cytogenetic alterations t(8;21) and inv(16) or t(16;16) were more frequent in younger patients with a median age of 15 years (IQR: 10–41) and 23 years (IQR: 12–48), respectively. Contrastingly, del(7q), ‐7, ‐5, ‐17, and del(5q) were associated with a median age above 55 years, with del(5q) at the upper end of the spectrum with a median age of 65 years (IQR: 55–71; Figure S1B). Cases of therapy‐associated AML (tAML) were rare, accounting for 3.4% (*n* = 54/1606) in the SAL cohort and 4.8% (*n* = 35/728) in the cohort initially published by Bottomly et al., while information on tAML status was missing for all pediatric cases, although the proportion of tAML is reportedly very low in this age group.^37^

### ML predicts patient outcome and quantifies prognostic impact

Three different ML models (RF, LR, and XGB) were compared for their predictive performance regarding CR and 2‐year OS prediction. For each model, 100 runs were performed. For CR prediction, the best performing model was RF with an average AUROC of 0.801 (range: 0.796–0.807; Figure S2A) and an average MCC of 0.446 (range: 0.421–0.477; Figure S2C). XGB achieved an average AUROC of 0.773 (range: 0.768–0.779; Figure S2A) and average MCC of 0.387 (range: 0.361–0.414; Figure S2C), while LR had an AUROC of 0.791 and MCC of 0.408 (since LR is a deterministic model, no ranges can be given). For the prediction of 2‐year OS, XGB outperformed RF and LR, achieving an average AUROC of 0.791 (range: 0.788–0.800; Figure S2B) and average MCC of 0.437 (range: 0.0.417–0.465; Figure S2D). RF showed an average AUROC of 0.769 (range: 0.764–0.775; Figure S2B) and average MCC of 0.421 (range: 0.393–0.440; Figure S2D), while LR had an AUROC of 0.780 and MCC of 0.402.

**Figure 2:**
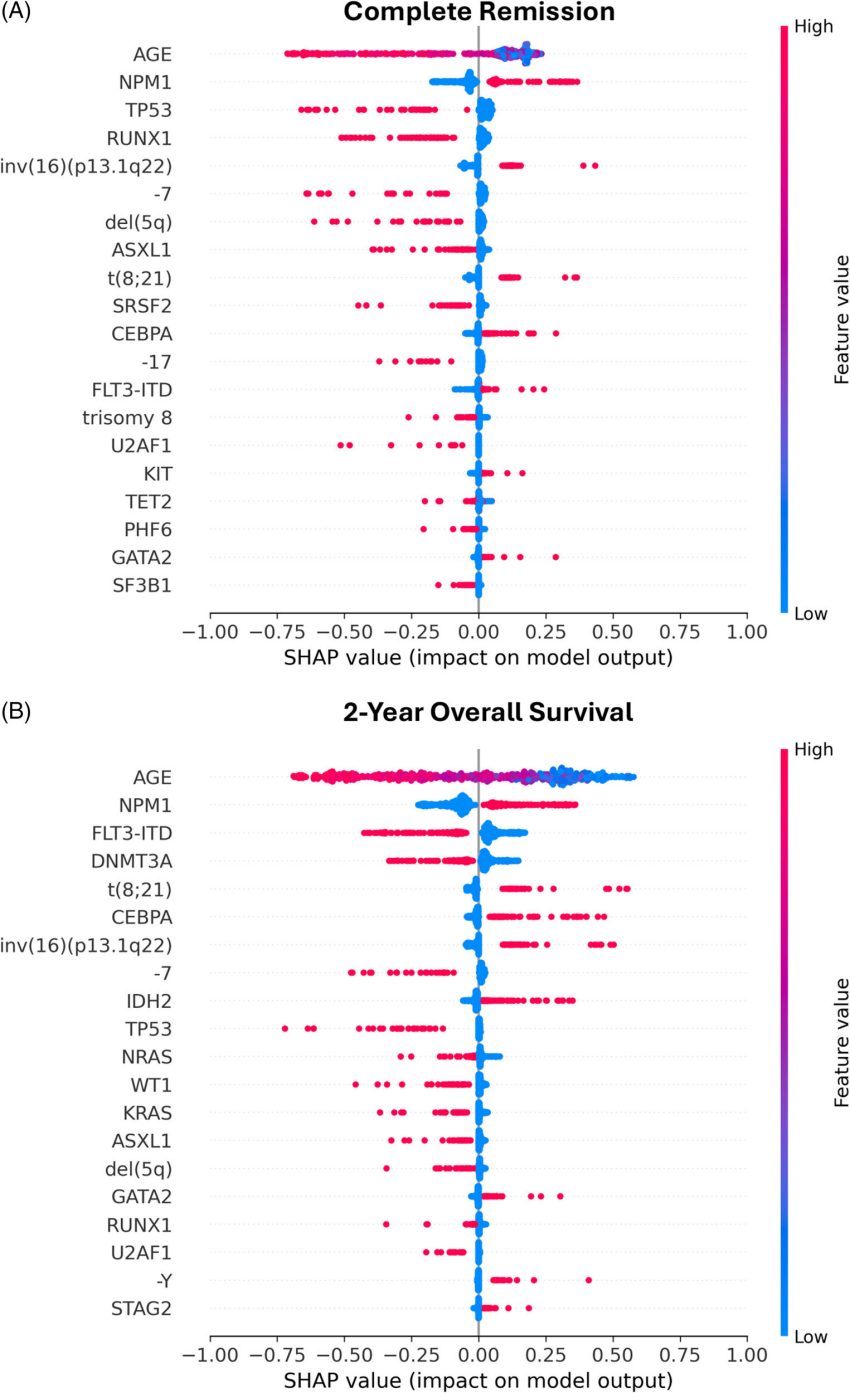
**SHAP beeswarm plot for prediction of complete remission after intensive induction therapy with Random Forest (A) and 2‐year overall survival with XGBoost (B).** The influence of individual variables on model predictions is displayed for the best performing models regarding complete remission (CR) (Random Forest; **A**) and 2‐year overall survival (OS) prediction (XGBoost; **B**). Automatically selected variables are listed from most influential (top) to least influential (bottom). SHAP values (*x*‐axis) represent the impact of each variable on model decisions. Positive SHAP values (>0) indicate a favorable prediction (i.e., achievement of CR), while negative values (<0) indicate an unfavorable prediction (i.e., treatment failure). Each single dot represents a single patient. Age was treated as a continuous variable. Hence, high feature values (red) represent older patients, while lower feature values (blue) represent younger patients. Genetic alterations were treated as binary variables: present/mutated (red) versus absent/wildtype (blue). The magnitude of the effect per patient is indicated by the SHAP value, with deviations from 0 showing either a positive (>0) or negative (<0) effect on treatment response. A positive impact on CR prediction **(A)** was found for alterations of *NPM1*, *CEBPA*, *FLT3*‐ITD, *KIT*, *GATA2*, inv(16), and t(8;21). Conversely, a negative impact (i.e., prediction of treatment failure) was observed for alterations of *TP53, RUNX1, ASXL1, SRSF2, U2AF1, TET2, PHF6*, and *SF3B1*, as well as ‐7, del(5q), ‐17, and trisomy 8. A positive impact on 2‐year OS prediction **(B)** was found for alterations of *NPM1, CEBPA, IDH2, GATA2*, and STAG2, as well as t(8;21), inv(16), and ‐Y. A negative impact on model predictions (i.e., death before the 2‐year mark) was observed for alterations of *DNMT3A, TP53, NRAS, WT1, KRAS, ASXL1, RUNX1*, *U2AF1*, and *FLT3‐*ITD, as well as ‐7 and del(5q).

An individual variable's influence on model decision was assessed using SHAP values. The impact on model decisions for the best performing model for CR prediction, RF, is shown in Figure 2A, where SHAP values > 0 indicate a positive impact on CR achievement and SHAP values < 0 indicate a negative impact on CR achievement (i.e., a higher likelihood of induction failure). Variables in Figure 2 are sorted from most influential (top) to least influential (bottom). Each single dot represents a single patient. We found age to be the most influential variable for our model. Higher feature values for age (red) were associated with unfavorable model predictions (SHAP < 0). However, a proportion of patients with higher age were also predicted to have favorable outcomes (SHAP > 0), while patients with younger age (blue dots) were predominately predicted to have favorable outcomes (SHAP > 0). Genetic alterations were treated as binary variables (0 = wildtype, 1 = mutated/altered). Hence, the presence of an alteration (red dots) either indicates treatment response (SHAP > 0) or treatment failure (SHAP < 0) with differing magnitudes. Absence of an alteration (wildtype) is indicated by blue dots. Achievement of CR was found to be more likely for patients with alterations of *NPM1*, *CEBPA*, *FLT3*‐ITD, *KIT*, *GATA2*, inv(16), and t(8;21). Treatment failure was associated with alterations of *TP53, RUNX1, ASXL1, SRSF2, U2AF1, TET2, PHF6*, and *SF3B1*, as well as ‐7, del(5q), ‐17, and trisomy 8. Beeswarm plots for XGB and LR regarding CR prediction are shown in Figures S3 and S4, respectively.

This analysis was repeated for prediction of 2‐year OS. Figure 2B shows the corresponding beeswarm plot for the best performing model in 2‐year OS prediction, which was XGB. Again, patient age was selected as the most influential variable in model decisions. As patient age decreased (blue), the likelihood of favorable outcomes (SHAP > 0) increased, and vice versa, as patient age increased (red), the likelihood of adverse outcomes (SHAP < 0) increased. However, in comparison to CR prediction (Figure 2A), heterogeneity in outcomes was more commonly indicated by a larger proportion of blue dots on the negative (%) and red dots on the positive sides (%) of the null. As for genetic alterations, *NPM1* was again selected as the most important genetic variable, with patients harboring an alteration being more likely to survive beyond the 2‐year mark. Interestingly, the effect of *FLT3*‐ITD was found to be reversed compared to CR prediction, as patients bearing the alteration were now more likely to experience adverse outcomes. Further variables associated with favorable outcomes were t(8;21), inv(16), and ‐Y, as well as alterations of *CEBPA, IDH2, GATA2*, and *STAG2*, while alterations of *DNMT3A, TP53, NRAS, WT1, KRAS, ASXL1, RUNX1*, and *U2AF1* as well as ‐7 and del(5q) were associated with higher risk. Beeswarm plots for RF and LR are shown in Figures S5 and S6, respectively.

### Genetic alterations and their impact on patient outcome are shaped by age groups

**Figure 3:**
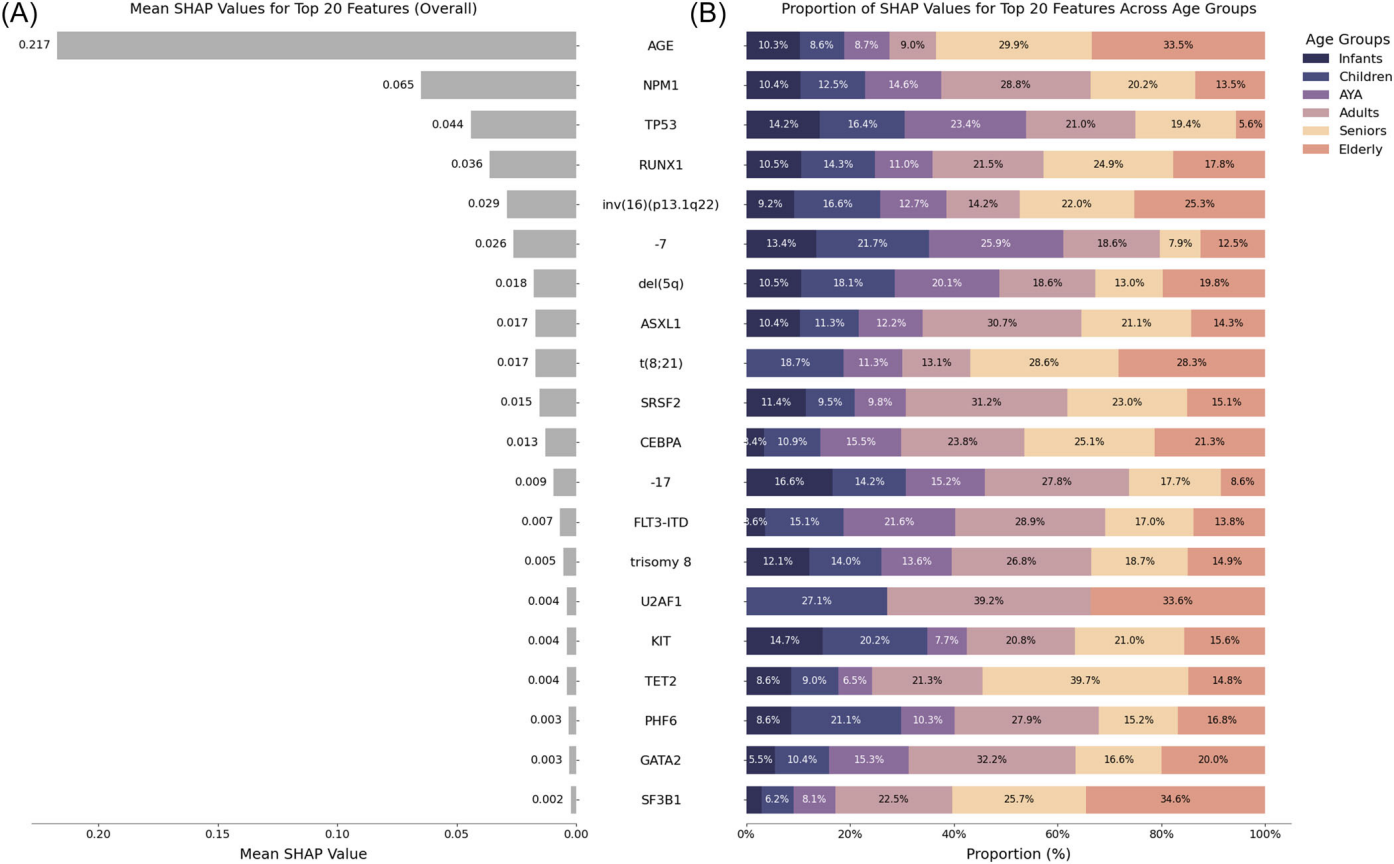
**Age‐group‐wise impact on model inference for prediction of complete remission with Random Forest.** For Random Forest (RF), individual variables impacting model decisions were ranked according to their mean SHAP values from highest to lowest **(A)**. The individual contributions for a given feature were traced back to the different age groups. The effect size derived from each age group is shown as percentage of the individual contribution per age group to the variable's mean SHAP value **(B)**, indicating differential prognostic impacts per age group for each single variable. We observed that no single variable had a uniform contribution to the overall SHAP values across all age groups, but rather that contributions to model explainability varied largely between age groups per variable.

To highlight differences in magnitude between individual variables' impact on RF's predictions for CR, we next compared mean SHAP values per variable for the overall cohort. Figure 3A ranks mean SHAP values for the most influential variables from top to bottom. Patient age was the most influential variable with a SHAP value of 0.217, surpassing genetic alterations. Among genetic alterations, *NPM1, TP53, RUNX1*, inv(16), and ‐7 had substantial influences on model decisions, while the remaining variables exhibited progressively smaller effects. Next, we analyzed the contributions of each variable to the overall SHAP values, broken down by age groups, to illustrate the differential impact of variables across age demographics. Figure 3B displays these differing contributions as percentages of mean SHAP values per variable and age group. For instance, patient age itself is highly influential on model decisions, as reflected by a large mean SHAP value (Figure 3A), which was primarily derived from contributions from patients in the senior and elderly groups, accounting for 29.9% and 33.5% of the overall effect, respectively (Figure 3B). For alterations of *NPM1*, the largest contributions to the mean SHAP value were derived from patients in the AYA (14.6%), adult (28.8%), and senior (20.2%) groups, consistent with age distributions for *NPM1* alterations (Figure 1). Interestingly, some alterations exhibited a contrasting pattern: Although alterations of *TP53* were more prevalent in older patients (Figure 1), their contribution to model decisions predominantly came from younger patient groups (children 16.4%, AYA 23.4%, adults 21.0%). A similar pattern was found for ‐7, del(5q), ‐17 and, though to a smaller degree, for alterations of *RUNX1* and *ASXL1*. Conversely, inv(16) and t(8;21) were more frequently found in younger patients (Figure S1), yet the highest contribution to mean SHAP values were observed for patients in the senior (inv(16): 22.0%; t(8;21): 28.6%) and elderly (inv(16): 25.3%; t(8;21): 28.3%) age groups. Mean SHAP values and age‐group‐wise contributions for CR prediction with XGB and LR are displayed in Figures S7 and S8, respectively.

**Figure 4:**
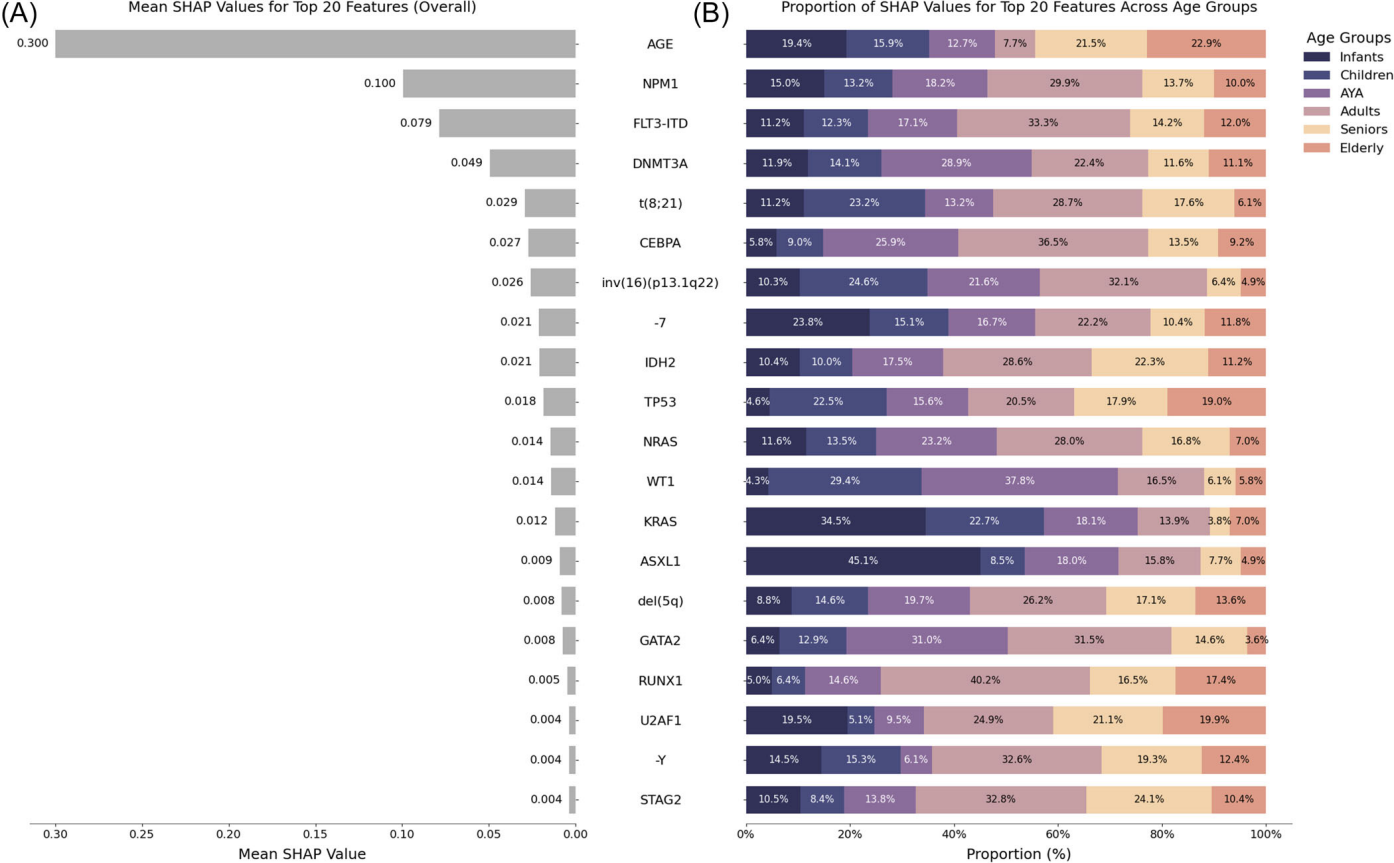
**Age‐group‐wise impact on model inference for prediction of 2‐year overall survival with XGBoost.** For XGBoost (XGB), individual variables impacting model decisions were ranked according to their mean SHAP values from highest to lowest **(A)**. The individual contributions for a given feature were traced back to the different age groups. The effect size derived from each age group is shown as percentage of the individual contribution per age group to the variable's mean SHAP value **(B)**, indicating differential prognostic impacts per age group for each single variable. Analogous to Figure 3, contributions to mean SHAP values were not uniform across age groups, indicating differing magnitudes of impact on model decisions per variable across all age groups.

For features selected for 2‐year OS, the influence of age surpassed that of genetic alterations by far, represented by a mean SHAP value of 0.300 (Figure 4A). Alterations of *NPM1* dominated the impact of genetic alterations with a mean SHAP value of 0.100, while *FLT3*‐ITD, alterations of *DNMT3A*, t(8;21), altered *CEBPA*, inv(16), and the remaining alterations had a progressively smaller impact on model decisions. The individual contributions to a variable's mean SHAP value were again analyzed per age group (Figure 4B). Patient age itself was most influential as a prognostic marker in infants (19.4% contribution to the mean SHAP value), children (15.9%), seniors (21.5%), and AYA (22.9%), while it played only a minor role in AYA and adults. For alterations of *NPM1*, the contributions to its mean SHAP value again were consistent with its age distribution (Figure 1). *FLT3*‐ITD, in contrast to CR prediction (Figure 2A) now an adverse risk marker for 2‐year OS (Figure 2B), also showed a consistent pattern with its age distribution (Figure 1). However, similar to the pattern observed for CR prediction, some genetic variables exhibited more pronounced effects (either as positive or negative risk markers) when found outside their age distribution. This was most pronounced for alterations of *ASXL1* deriving 45.1% of its mean SHAP value only from patients (2.4% [*n* = 3] of 126 infant patients) in the infant group (Figure 4B), while its median age exceeded 60 years (Figure 1). Similar effects were found for ‐7, where 55.6% of its mean SHAP value came from infants, children, and AYA, similar to del(5q) with 43.1% and alterations of *U2AF1* with 34.1%, even though these alterations typically occurred in individuals with a median age beyond 60 years (Figures 1 and S1). Mean SHAP values and age‐group‐wise contributions for 2‐year OS prediction with RF and LR are displayed in Figures S9 and S10, respectively.

In subgroup analysis, we dichotomized for female (47.0%, *n* = 1439) and male (53.0%, *n* = 1623) patients within our cohorts. For CR prediction, RF showed better performance in male patients with an AUROC of 0.82 and an MCC of 0.47 compared to female patients who had an AUROC of 0.78 and an MCC of 0.42. Similarly, for 2‐year OS prediction, XGBoost (XGB) performed better in male patients, achieving an AUROC of 0.79 and an MCC of 0.46, compared to an AUROC of 0.74 and an MCC of 0.36 in female patients. In both CR and 2‐year OS predictions, age remained the most influential variable regardless of sex. Regarding genetic alterations, we found *NPM1*, *RUNX1*, ‐7, *TP53*, del(5q), and *ASXL1* to be most influential for CR prediction in female patients (Figure S11), while *NPM1*, *RUNX1*, *TP53*, inv(16), *SRSF2*, and *CEBPA* were the most influential variables for male patients (Figure S12). For 2‐year OS, the most influential genetic variables in female patients (Figure S13) were *FLT3*‐ITD, *NPM1*, *DNMT3A*, *CEBPA*, *IDH2*, and t(8;21), while for male patients (Figure S14) *NPM1*, *FLT3*‐ITD, *TP53*, inv(16), *CEBPA*, and ‐7 were most influential on model decisions. Notably, no individual genetic variable had a consistent effect across all age groups. Instead, the effect sizes varied by age.

### Age‐dependency of prognostic markers is confirmed in univariable analyses

**Figure 5:**
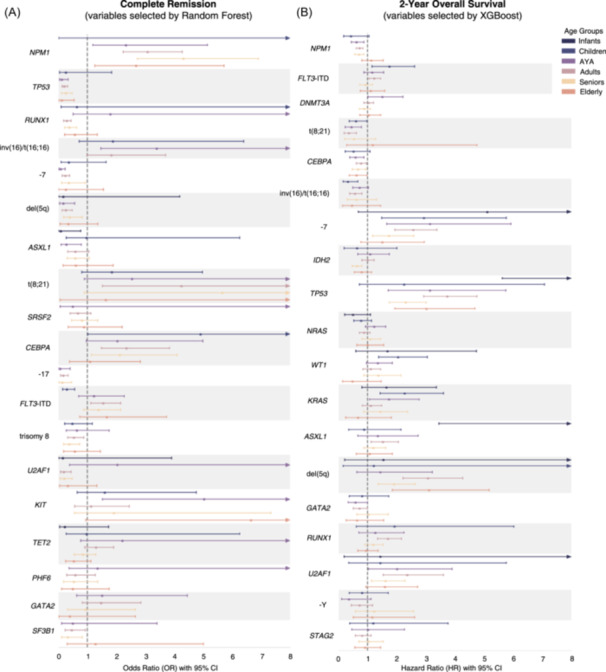
**Forest plots for univariable analyses of genetic alterations per age group.** Univariable analyses per age group for complete remission **(A)** and 2‐year OS **(B)** regarding genetic alterations that were previously selected by machine learning models confirmed the age‐dependency reflected in SHAP values. No single alteration was uniformly associated with favorable or adverse effects, rather individual alterations were found to be prognostic depending on the assignment of the patient bearing the alteration to one of the age groups. Odds ratios (OR) and hazard ratios (HR) are displayed as point estimates with 95% confidence intervals (CI). Arrowheads indicate that the upper limit of the CI is out of bounds of the plot. If a combination of genetic alteration and age group had a non‐significant effect with an OR/HR out of bounds of the plot, it is not shown. Patient numbers as well as numerical values for OR/HR, 95% CI, *p* values, and adjusted *p* values are displayed in Tables S4 and S5 for CR and 2‐year OS, respectively.

To confirm the age‐dependency of the observed variables selected by our models for CR and 2‐year OS predictions, we next conducted univariable analyses for each alteration per age group. OR and HR for achievement of CR and 2‐year OS with 95% CI are visualized in Figure 5. Detailed numerical values for OR/HR, 95% CI, *p* values, adjusted *p* values, as well as the number of patients per age group and variable are shown in Tables S4 and S5. Generally, for both CR and 2‐year OS, we observed the impact of genetic alterations to be dependent on age groups, as no single alteration exhibited a uniform favorable or adverse effect across all six age groups (Figure 5). After adjusting for multiple hypotheses testing using the method by Benjamini and Hochberg,^33^ favorable treatment response (i.e., significantly higher odds of achieving CR) was only found for adult and senior patients with *NPM1* alterations, children with *FLT3*‐ITD, and adult patients with *CEBPA* alterations. Conversely, treatment failures (i.e., significantly lower odds of achieving CR) were observed in AYA, adults, and seniors with altered *TP53*, adults and seniors with altered *RUNX1* and *U2AF1*, as well as AYA and adults with either ‐7 or del(5q), and adults with ‐17 (Table S4). Regarding 2‐year OS, favorable outcomes, i.e., significantly lower HRs, were found for AYA, adults, and seniors with alterations of *NPM1*, seniors with alterations of *IDH2*, children and adults with inv(16)/t(16;16), as well as AYA and adults with t(8;21). Adverse risk (i.e., significantly higher HRs) was observed for infants, AYA, adults, seniors, and elderly with *TP53* alterations, infants and adults with altered *ASXL1*, children with alterations in either *WT1, KRAS*, or *FLT3*‐ITD, adults and seniors with *U2AF1* alterations, adults with *RUNX1* alterations, ‐7 in children, AYA, adults, and seniors, and del(5q) in adults, seniors, and elderly.

## DISCUSSION

Current treatment recommendations, such as ELN2022,^10^ function essentially as decision trees, assuming that once a genetic variable is identified, its impact on personalized risk is context‐insensitive. This implies that an alteration has a fixed effect irrespective of patient context, with limited consideration of co‐occurring risk‐modifying alterations. Given the simplicity of this model, designed for practical clinical decision‐making, we hypothesized that incorporating patient age with genetic alterations could offer additional insights into risk modification.

In a large multicenter cohort of both pediatric and adult AML patients, we identified substantial differences in the prevalence of genetic alterations affecting pathways and cell functions between younger and older patients. Alterations in signaling pathways were more commonly found in younger patients, whereas alterations in epigenetic regulators, the spliceosome, and *TP53* increased with age, consistent with previous reports.^13^, ^38^, ^39^ We trained three ML models to predict CR and 2‐year OS and extract prognostically relevant variables. With an AUROC of 0.801, RF achieved the best performance of our models for CR prediction, while XGB outperformed RF and LR with an AUROC of 0.773 in predicting 2‐year OS. Several recent studies also demonstrated ML's capability of accurately predicting patient outcomes in AML leveraging a wealth of available patient data from clinical trials, genetic testing, and knowledge banks, albeit none of these studies combined pediatric and adult patients.^40^, ^41^, ^42^, ^43^, ^44^, ^45^ To make model decisions explainable, we used SHAP values quantifying the impact of a variable on model outputs. The magnitude of the prognostic effect was traced back to age groups, unveiling differential contributions to model decisions depending on the age‐related context in which a genetic alteration was found. Previous studies usually dichotomized adult patients into younger and older at thresholds of 60, 65, or 75 years of age, reporting differential prognostic effects for alterations such as *FLT3*‐ITD, *NPM1*, *DNMT3A*, and *TP53*, among others.^46^, ^47^, ^48^, ^49^ Our model further differentiates between six age groups, indicating varying effects of differing magnitudes per genetic variable. In line with previous studies, alterations of *NPM1* were a top contributor to positive model decisions for both CR and 2‐year OS. The positive prognostic impact of *NPM1* mutations are well described in adult AML,^50^ while *NPM1* mutations in children are rare,^51^ although similar favorable outcomes^52^ have been reported. However, recent reports suggest that these favorable effects do not necessarily extend to older patients^53^, ^54^ and are further abrogated in individuals with co‐occurring mutations in *DNMT3A* and *FLT3*‐ITD.^55^ The prevalence of both *DNMT3A* and *RUNX1* alterations increases with age and an unfavorable prognostic impact has been described across all age groups.^48^, ^56^, ^57^, ^58^, ^59^, ^60^, ^61^ A similarly uniform but positive prognostic impact has been reported for core binding factor AML,^62^ showing high CR rates even in older patients receiving intensive treatment while OS decreases with age.^63^ While the majority of autonomously selected variables in our models are in accordance with ELN recommendations^10^ regarding effect directionality, there were two outliers: *FLT3*‐ITD influenced model decisions positively toward CR achievement, while it had a negative impact on overall survival. Recently, studies showed a differing impact of *FLT3*‐ITD dependent on patient age^64^ and co‐occurring alterations,^65^ while high risk disease was reported for a subgroup of patients with altered *NPM1*, *DNMT3A*, and *FLT3*‐ITD.^55^ Further, alterations of *GATA2* contributed toward both increased odds of treatment response and better overall survival. A recent meta‐analysis of 13 cohorts^66^ found alterations of *GATA2* to be associated with poor outcomes in MDS, but not in AML. Previous reports suggest an adverse effect of high levels of *GATA2* expression in AML.^67^, ^68^ This highlights the need to approach risk stratification in a more nuanced manner contrary to current simplified binary risk allocation models: A genetic alteration can predict prognosis depending on the context in which it is found, not merely on its absence or presence. The frequency with which a genetic alteration occurred expectedly differed with age. However, the risk associated with the specific alteration was occasionally found to be disassociated from its age distribution. This may reflect patterns associated with higher risk disease and may prompt closer monitoring of younger AML patients with aging‐related patterns. Additionally, data‐driven analysis with ML models addresses a key limitation of conventional univariable analysis, which relies on manual a priori variable selection and often fails to capture complex, nonlinear relationships between features. This capability becomes increasingly crucial as affordable genetic sequencing and omics techniques become more integrated into clinical practice.

A notable limitation of our study is the restriction to genetic alterations covered by the myeloid panel used in the SAL studies, which was necessary to merge all available datasets. This highlights the trade‐off between the granularity of genetic information and the applicability of ML models, which require large datasets for training. However, WGS/WES data were not available for the SAL cohort, necessitating a compromise by focusing on alterations covered in the myeloid panel. Nevertheless, these alterations comprise the most commonly used items in routine diagnostics, allowing for a widespread implementation of our findings. Importantly, models such as ELN generate mutually exclusive patient subgroups based on predefined (evidence‐based) assumptions of risk associated with certain variables (and therefore essentially function as manual clustering). Clustering frameworks such as the ELN model allow for straightforward comparisons between versions (e.g., ELN2017 vs. ELN2022) through Kaplan–Meier or Sankey analyses, as patients can be easily (and manually) assigned to groups. In contrast, our supervised models start by predicting clinical outcomes (CR, 2‐year OS) and retrospectively determine which variables contributed most to those predictions, allowing for a more context‐sensitive evaluation of risk factors on a patient‐level. Given this fundamental methodological difference, direct performance comparisons between our supervised approach and clustering frameworks such as ELN's model are methodologically not directly comparable; however, their results may complement each other. Moreover, 15.9% of patients in the cohort derived from Bottomly et al.^16^ were treated with non‐intensive therapy, while all other patients including those in the SAL, TARGET, and Fred Hutch cohort were treated with intensive chemotherapy. Balancing homogeneity to reduce potential confounding with the heterogeneity required to ensure generalizability and sufficient sample size is a well‐known challenge in medical research, particularly for rare diseases such as AML. Increasing the sample size may introduce the possibility of further substratification by treatment modalities such as type of intensive induction therapy without risking stability in the training process or generalizability of results in a limited sample as ours. A more comprehensive analysis, ideally covering a broader range of genetic alterations and gene expression data, is desirable to further characterize the interplay between age, AML biology, and patient outcomes, also extending to different treatment regimens (intensive, non‐intensive, targeted agents) as an adjustable variable to potentially predict how an individual patient may fare given a certain therapy. Ideally, this may also include predicting other clinically relevant endpoints such as MRD negativity or relapse‐free survival. Further, information on comorbidities influencing patient outcomes and potentially modulating disease biology or limiting survival should be incorporated when available. This is particularly relevant for older patients, as comorbidities that limit overall survival are expected to be more prevalent and, potentially, contribute more strongly to disease and outcome modification. To this end, international collaborative efforts in data collection and harmonization are needed to provide a representative database for adaptive risk stratification model development, as manual feature selection in current models in clinical routine likely underestimates inter‐variable connections. For routine clinical applicability, future models need to seamlessly integrate into routine workflows without posing additional manual labor to healthcare providers. Integration in clinical information systems and electronic health records may be an option if patient privacy is preserved. In line with this, our analysis represents only a step forward from previous studies that dichotomized age, whereas we use six age groups entailing both pediatric and adult AML. While one could argue for different age group thresholds, no single cut‐off accurately reflects biological variation. We selected our age group thresholds based on accepted cut‐offs from academic societies and the scientific literature to capture as much heterogeneity as possible.^28^, ^29^, ^30^, ^31^, ^32^ Future work may include subgroup analysis for different treatment modalities such as ASCT.

In summary, our study underscores the need for improved risk stratification in AML by contextualizing prognostic genetic alterations selected by highly accurate ML models predicting both CR and 2‐year OS. Our findings not only improve upon the risk stratification for individual genetic variables but also leave simplified age dichotomizations behind and prompt a close monitoring of younger patients with genetics related to aging.

## AUTHOR CONTRIBUTIONS

**Jan‐Niklas Eckardt**: Conceptualization (lead); data curation (equal); formal analysis (lead); investigation (lead); methodology (lead); project administration (lead); resources (equal); software (equal); validation (equal); visualization (equal); writing—original draft preparation (lead). **Waldemar Hahn**: Data curation (equal); formal analysis (equal); investigation (equal); methodology (equal); software (lead); validation (equal); visualization (equal); writing—review and editing (equal). **Rhonda E. Ries**: Data curation (equal); formal analysis (equal); investigation (equal); methodology (equal); resources (equal); validation (equal); writing—review and editing (equal). **Szymon D. Chrost**: Data curation (equal); formal analysis (equal); investigation (equal); methodology (equal); software (lead); validation (equal); visualization (equal); writing—review and editing (equal). **Susann Winter**: Formal analysis (equal); investigation (equal); project administration (equal); validation (equal); writing—review and editing (equal). **Sebastian Stasik**: Data curation (equal); formal analysis (equal); investigation (equal); methodology (equal); resources (equal); validation (equal); writing—review and editing (equal). **Christoph Röllig**: Formal analysis (equal); investigation (equal); resources (equal); validation (equal); writing—review and editing (equal). **Uwe Platzbecker**: Formal analysis (equal); investigation (equal); resources (equal); validation (equal); writing—review and editing (equal). **Carsten Müller‐Tidow**: Formal analysis (equal); investigation (equal); resources (equal); validation (equal); writing—review and editing (equal). **Hubert Serve**: Formal analysis (equal); investigation (equal); resources (equal); validation (equal); writing—review and editing (equal). **Claudia D. Baldus**: Formal analysis (equal); investigation (equal); resources (equal); validation (equal); writing—review and editing (equal). **Christoph Schliemann**: Formal analysis (equal); investigation (equal); resources (equal); validation (equal); writing—review and editing (equal). **Kerstin Schäfer‐Eckart**: Formal analysis (equal); investigation (equal); resources (equal); validation (equal); writing—review and editing (equal). **Maher Hanoun**: Formal analysis (equal); investigation (equal); resources (equal); validation (equal); writing—review and editing (equal). **Martin Kaufmann**: Formal analysis (equal); investigation (equal); resources (equal); validation (equal); writing—review and editing (equal). **Andreas Burchert**: Formal analysis (equal); investigation (equal); resources (equal); validation (equal); writing—review and editing (equal). **Johannes Schetelig**: Formal analysis (equal); investigation (equal); resources (equal); validation (equal); writing—review and editing (equal). **Martin Bornhäuser**: Formal analysis (equal); investigation (equal); resources (equal); validation (equal); writing—review and editing (equal). **Markus Wolfien**: Data curation (equal); formal analysis (equal); investigation (equal); methodology (equal); software (supporting); validation (equal); writing—review and editing (equal). **Soheil Meshinchi**: Conceptualization (equal); formal analysis (equal); funding acquisition (equal); investigation (equal); resources (equal); supervision (equal); validation (equal); writing—review and editing (equal). **Christian Thiede**: Conceptualization (equal); formal analysis (equal); funding acquisition (equal); investigation (equal); resources (equal); supervision (equal); validation (equal); writing—review and editing (equal). **Jan Moritz Middeke**: Conceptualization (equal); formal analysis (equal); funding acquisition (equal); investigation (equal); resources (equal); supervision (equal); validation (equal); writing—review and editing (equal).

## CONFLICT OF INTEREST STATEMENT

The authors declare no conflicts of interest.

## FUNDING

J.‐N.E. received a clinical fellowship via the Mildred Scheel Early Career Center (MSNZ) funded by the Mildred Scheel foundation (Deutsche Krebshilfe).

## Supporting information

Supporting information.

## Data Availability

Data pertaining to the SAL study group cohort and Seattle cohort can be inquired from the corresponding author upon reasonable request. The results published here are in part based upon publicly available data generated by the Therapeutically Applicable Research to Generate Effective Treatments (https://www.cancer.gov/ccg/research/genome-sequencing/target) initiative, phs000218, specifically the AML subgroup (phs000465), and data available at the Genomic Data Commons (https://portal.gdc.cancer.gov).
