# Supplementary material for: Age‐stratified machine learning identifies divergent prognostic significance of molecular alterations in AML
Source: Hemasphere. 2025 May 7;9(5):e70132. doi: 10.1002/hem3.70132 (PMC12056602; doi:10.1002/hem3.70132)
Supplement: Supplementary file 1 — Supporting information. [file HEM3-9-e70132-s001.docx]

# Supplemental Tables

## Supplemental Table S1. Summary of trial regimens for the Study Alliance Leukemia (SAL) cohort

| **trial name** | **clinicaltrials.gov identifier** | **trial duration** | **protocol summary** |
| --- | --- | --- | --- |
| AML96 | NCT00180115 | 1996-2008 | risk-adapted postremission treatment regarding allogeneic stem cell transplantation for high-risk AML and related allogeneic and autologous stem cell transplantation for standard-risk AML, and randomization between intermediate-dose and high-dose cytarabine within the first post-remission course |
| AML2003 | NCT00180102 | 2003-2009 | early allogeneic stem cell transplantation in post-induction aplasia for high-risk AML, factorial design with four therapy arms with two factors of two stages (intensified vs. standard therapy and cytarabine vs. cytarabine + mitoxantrone + amsacrin) |
| AML60+ | NCT00180167 | 2005-2010 | Patients ≥ 60 years, mitoxantron on day 1,2,3 + cytarabine on days 1,3,5,7 vs. DA 7+3 |
| SORAML | NCT00893373 | 2011-2014 | Standard therapy + sorafenib vs. standard therapy + placebo |

## Supplemental Table S2. Baseline patient characteristics per cohort.

|  | **SAL** | **Fred Hutch** | **Bottomly** | **TARGET** |
| --- | --- | --- | --- | --- |
| **n of patients** | 1606 | 578 | 728 | 150 |
| infants (0-2 y) | 0 | 94 | 0 | 29 |
| children (3-14y) | 0 | 318 | 0 | 77 |
| AYA (15-39y) | 274 | 166 | 105 | 44 |
| adults (40-64y) | 898 | 0 | 322 | 0 |
| seniors (65-74y) | 318 | 0 | 200 | 0 |
| elderly (75y+) | 116 | 0 | 101 | 0 |
| **age (years), median (IQR)** | 56 (44-65) | 11 (5-15) | 62 (48-71) | 10 (3-15) |
| **sex, n (%)** |  |  |  |  |
| female | 768 (47.8) | 275 (47.6) | 323 (44.4) | 73 (48.7) |
| male | 838 (52.2) | 303 (52.4) | 405 (55.6) | 77 (51.3) |
| **de novo AML, n (%)** |  |  |  |  |
| yes | 1339 (83.4) | n.a. | 365 (50.1) | n.a. |
| no | 249 (15.5) | n.a. | 362 (49.9) | n.a. |
| **laboratory, median (IQR)** |  |  |  |  |
| WBC (10^9^/l) | 19.0 (4.5-53.4) | 39.4 (12.7-94.5) | n.a. | 44.8 (14.9-112.5) |
| Hb (mmol/l) | 5.9 (5.0-7.0) | n.a. | n.a. | n.a. |
| Plt (10^9^/l) | 50 (27-94) | n.a. | n.a. | n.a. |
| PBB (%) | 40 (12-73) | 49 (20-77) | n.a. | 62 (35-83) |
| BMB (%) | 63 (44-79) | 71 (50-86) | n.a. | 75 (59-90) |
| **cytogenetics** |  |  |  |  |
| **normal karyotype, n (%)** |  |  |  |  |
| yes | 829 (51.6) | 140 (24.2) | 284 (39.0) | 27 (18.0) |
| no | 668 (41.6) | 415 (72.0) | 379 (52.1) | 113 (75.3) |
| **complex karyotype, n (%)** |  |  |  |  |
| yes | 184 (11.5) | 62 (10.7) | 140 (19.2) | 16 (10.7) |
| no | 1320 (82.2) | 493 (85.3) | 523 (71.8) | 124 (82.7) |
| **non-intensive therapy, n (%)** | 0 | 0 | 116 (15.9) | 0 |
| **allo HSCT, n (%)** |  |  |  |  |
| yes | 519 (32.3) | 78 (13.5) | 232 (31.9) | 13 (8.7) |
| no | 1087 (67.7) | 458 (79.2) | 496 (68.1) | 137 (91.3) |

Abbreviations: acute myeloid leukemia (AML), allogeneic (allo), bone marrow blasts (BMB), hemoglobin (Hb), hematopoietic stem cell transplantation (HSCT), interquartile range (IQR), not available (n.a.), number (n/N), peripheral blood blasts (PBB), platelet count (Plt), white blood cell count (WBC).

## Supplemental Table S3. Myeloid Gene Panel

Summary of the 54 genes targeted by the TruSight Myeloid Sequencing Panel (Illumina, San Diego, CA, USA).

| TruSight Myeloid Sequencing Panel | | | | |
| --- | --- | --- | --- | --- |
| *ABL1* | *CEBPA* | *HRAS* | *MYD88* | *SF3B1* |
| *ASXL1* | *CSF3R* | *IDH1* | *NOTCH1* | *SMC1A* |
| *ATRX* | *CUX1* | *IDH2* | *NPM1* | *SMC3* |
| *BCOR* | *DNMT3A* | *IKZF1* | *NRAS* | *SRSF2* |
| *BCORL1* | *ETV6/TEL* | *JAK2* | *PDGFRA* | *STAG2* |
| *BRAF* | *EZH2* | *JAK3* | *PHF6* | *TET2* |
| *CALR* | *FBXW7* | *KDM6A* | *PTEN* | *TP53* |
| *CBL* | *FLT3* | *KIT* | *PTPN11* | *U2AF1* |
| *CBLB* | *GATA1* | *KRAS* | *RAD21* | *WT1* |
| *CBLC* | *GATA2* | *MLL* | *RUNX1* | *ZRSR2* |
| *CDKN2A* | *GNAS* | *MPL* | *SETBP1* |  |

## Supplemental Table S4. Univariable analyses of genetic alterations per age group for achievement of complete remission

| **Variable** | **Age Group** | **Pts. mut.** | **Pts. w. info.** | **OR** | **lower CI** | **upper**  **CI** | ***p*** | ***p adj.*** |
| --- | --- | --- | --- | --- | --- | --- | --- | --- |
| *NPM1* | infants | 0 | 125 |  |  |  |  |  |
|  | children | 29 | 401 | 6003256 | 6.17E-10 | 9.72E+98 | 0.983 | 1.000 |
|  | AYA | 119 | 575 | 2.33 | 1.19 | 5.13 | **0.022** | 0.171 |
|  | adults | 405 | 1154 | 3.06 | 2.23 | 4.28 | **1.44E-11** | **4E-09** |
|  | seniors | 119 | 439 | 4.30 | 2.73 | 6.91 | **7E-10** | **6.49E-08** |
|  | elderly | 40 | 141 | 2.67 | 1.27 | 5.72 | **0.010** | 0.102 |
| *TP53* | infants | 1 | 125 | 914792.7 | 1.5E-123 |  | 0.992 | 0.998 |
|  | children | 6 | 403 | 0.25 | 0.05 | 1.84 | 0.115 | 0.464 |
|  | AYA | 14 | 576 | 0.11 | 0.04 | 0.34 | **8.78E-05** | **0.002** |
|  | adults | 75 | 1162 | 0.20 | 0.12 | 0.32 | **5.85E-11** | **8.13E-09** |
|  | seniors | 53 | 451 | 0.24 | 0.12 | 0.47 | **5.81E-05** | **0.001** |
|  | elderly | 14 | 154 | 0.11 | 0.01 | 0.55 | 0.033 | 0.203 |
| *RUNX1* | infants | 0 | 125 |  |  |  |  |  |
|  | children | 6 | 403 | 0.64 | 0.10 | 12.39 | 0.686 | 0.998 |
|  | AYA | 23 | 576 | 1.78 | 0.51 | 11.24 | 0.444 | 0.900 |
|  | adults | 92 | 1162 | 0.29 | 0.19 | 0.45 | **1.86E-08** | **1.29E-06** |
|  | seniors | 73 | 451 | 0.37 | 0.21 | 0.63 | **0.000** | **0.006** |
|  | elderly | 28 | 154 | 0.57 | 0.22 | 1.35 | 0.216 | 0.608 |
| inv(16)/  t(16;16) | infants | 18 | 118 | 23687102 | 4.34E-36 |  | 0.991 | 0.998 |
|  | children | 60 | 392 | 1.86 | 0.71 | 6.39 | 0.253 | 0.646 |
|  | AYA | 95 | 543 | 3.39 | 1.46 | 9.88 | **0.011** | 0.102 |
|  | adults | 71 | 1094 | 1.82 | 0.98 | 3.70 | 0.074 | 0.363 |
|  | seniors | 7 | 413 | 6610822 | 2.46E-15 |  | 0.977 | 0.998 |
|  | elderly | 4 | 136 | 27389881 | 5.37E-53 |  | 0.989 | 0.998 |
| -7 | infants | 1 | 118 | 2.75E-08 |  | 1.7E+121 | 0.990 | 0.998 |
|  | children | 12 | 392 | 0.35 | 0.10 | 1.64 | 0.130 | 0.481 |
|  | AYA | 12 | 543 | 0.07 | 0.02 | 0.24 | **3.03E-05** | **0.001** |
|  | adults | 61 | 1094 | 0.23 | 0.14 | 0.39 | **4.85E-08** | **2.7E-06** |
|  | seniors | 20 | 413 | 0.35 | 0.11 | 0.93 | 0.048 | 0.254 |
|  | elderly | 7 | 136 | 0.25 | 0.01 | 1.55 | 0.212 | 0.608 |
| del(5q) | infants | 2 | 118 | 0.16 | 0.01 | 4.18 | 0.203 | 0.594 |
|  | children | 3 | 392 | 715566.3 | 1.82E-42 |  | 0.987 | 0.998 |
|  | AYA | 10 | 543 | 0.15 | 0.04 | 0.57 | **0.004** | **0.049** |
|  | adults | 40 | 1094 | 0.25 | 0.13 | 0.47 | **0.000** | **0.001** |
|  | seniors | 36 | 413 | 0.39 | 0.18 | 0.82 | 0.016 | 0.136 |
|  | elderly | 11 | 136 | 0.33 | 0.05 | 1.36 | 0.171 | 0.580 |
| *ASXL1* | infants | 3 | 125 | 0.07 | 0.00 | 0.77 | 0.034 | 0.203 |
|  | children | 17 | 403 | 0.96 | 0.26 | 6.25 | 0.963 | 0.998 |
|  | AYA | 16 | 576 | 0.26 | 0.09 | 0.79 | **0.012** | 0.104 |
|  | adults | 52 | 1162 | 0.57 | 0.32 | 1.04 | 0.062 | 0.317 |
|  | seniors | 47 | 451 | 0.57 | 0.30 | 1.06 | 0.081 | 0.387 |
|  | elderly | 14 | 154 | 0.60 | 0.16 | 1.89 | 0.407 | 0.864 |
| t(8;21) | infants | 1 | 117 | 988715.3 | 1.6E-123 |  | 0.992 | 0.998 |
|  | children | 86 | 392 | 1.83 | 0.80 | 4.97 | 0.186 | 0.594 |
|  | AYA | 47 | 542 | 2.54 | 0.90 | 10.65 | 0.127 | 0.481 |
|  | adults | 41 | 1094 | 4.24 | 1.52 | 17.65 | **0.017** | 0.137 |
|  | seniors | 6 | 413 | 5.65 | 0.90 | 108.84 | 0.115 | 0.464 |
|  | elderly | 2 | 136 | 1.63 | 0.06 | 41.76 | 0.733 | 0.998 |
| *SRSF2* | infants | 0 | 125 |  |  |  |  |  |
|  | children | 0 | 403 |  |  |  |  |  |
|  | AYA | 4 | 576 | 0.49 | 0.06 | 10.07 | 0.545 | 0.998 |
|  | adults | 72 | 1162 | 0.66 | 0.40 | 1.12 | 0.112 | 0.464 |
|  | seniors | 62 | 451 | 0.80 | 0.46 | 1.37 | 0.417 | 0.873 |
|  | elderly | 22 | 154 | 0.88 | 0.33 | 2.20 | 0.787 | 0.998 |
| *CEBPA* | infants | 1 | 125 | 914792.7 | 1.5E-123 |  | 0.992 | 0.998 |
|  | children | 36 | 403 | 4.89 | 1.02 | 87.96 | 0.122 | 0.481 |
|  | AYA | 85 | 573 | 2.02 | 0.96 | 4.98 | 0.089 | 0.404 |
|  | adults | 159 | 1144 | 2.34 | 1.49 | 3.84 | **0.000** | **0.006** |
|  | seniors | 46 | 431 | 2.11 | 1.13 | 4.09 | **0.022** | 0.171 |
|  | elderly | 20 | 130 | 1.08 | 0.39 | 2.83 | 0.878 | 0.998 |
| -17 | infants | 0 | 118 |  |  |  |  |  |
|  | children | 0 | 392 |  |  |  |  |  |
|  | AYA | 4 | 543 | 0.05 | 0.00 | 0.41 | **0.011** | 0.102 |
|  | adults | 29 | 1094 | 0.16 | 0.07 | 0.34 | **0.000** | **0.000** |
|  | seniors | 18 | 413 | 0.13 | 0.02 | 0.46 | **0.007** | 0.080 |
|  | elderly | 6 | 136 | 9.58E-08 |  | 5.54E+33 | 0.987 | 0.998 |
| *FLT3*-ITD | infants | 1 | 125 | 914792.7 | 1.5E-123 |  | 0.992 | 0.998 |
|  | children | 63 | 403 | 0.28 | 0.15 | 0.57 | **0.000** | **0.005** |
|  | AYA | 129 | 575 | 1.23 | 0.70 | 2.27 | 0.494 | 0.974 |
|  | adults | 285 | 1155 | 1.54 | 1.12 | 2.15 | **0.010** | 0.102 |
|  | seniors | 100 | 446 | 1.37 | 0.88 | 2.15 | 0.165 | 0.567 |
|  | elderly | 31 | 142 | 1.66 | 0.74 | 3.74 | 0.214 | 0.608 |
| trisomy 8 | infants | 10 | 118 | 7947932 | 1.86E-35 |  | 0.990 | 0.998 |
|  | children | 42 | 392 | 0.47 | 0.21 | 1.17 | 0.082 | 0.387 |
|  | AYA | 30 | 543 | 0.63 | 0.26 | 1.75 | 0.333 | 0.799 |
|  | adults | 73 | 1094 | 0.52 | 0.32 | 0.87 | **0.011** | 0.102 |
|  | seniors | 44 | 413 | 0.38 | 0.18 | 0.74 | **0.006** | 0.071 |
|  | elderly | 22 | 136 | 0.55 | 0.19 | 1.46 | 0.252 | 0.646 |
| *U2AF1* | infants | 2 | 125 | 0.15 | 0.01 | 3.91 | 0.187 | 0.594 |
|  | children | 4 | 403 | 750309.9 | 4.52E-31 |  | 0.985 | 0.998 |
|  | AYA | 13 | 576 | 2.02 | 0.39 | 36.99 | 0.503 | 0.978 |
|  | adults | 22 | 1162 | 0.19 | 0.07 | 0.44 | **0.000** | **0.003** |
|  | seniors | 30 | 451 | 0.20 | 0.06 | 0.48 | **0.001** | **0.016** |
|  | elderly | 11 | 154 | 0.33 | 0.05 | 1.32 | 0.161 | 0.566 |
| *KIT* | infants | 9 | 125 | 7305675 | 8.82E-40 |  | 0.990 | 0.998 |
|  | children | 63 | 403 | 1.59 | 0.66 | 4.75 | 0.348 | 0.821 |
|  | AYA | 57 | 576 | 5.01 | 1.52 | 30.98 | **0.027** | 0.194 |
|  | adults | 43 | 1162 | 1.13 | 0.57 | 2.44 | 0.744 | 0.998 |
|  | seniors | 11 | 451 | 1.90 | 0.57 | 7.34 | 0.312 | 0.760 |
|  | elderly | 5 | 154 | 6.64 | 0.95 | 131.71 | 0.094 | 0.422 |
| *TET2* | infants | 5 | 125 | 0.21 | 0.03 | 1.73 | 0.106 | 0.456 |
|  | children | 17 | 403 | 0.96 | 0.26 | 6.25 | 0.963 | 0.998 |
|  | AYA | 41 | 576 | 2.19 | 0.77 | 9.24 | 0.199 | 0.594 |
|  | adults | 196 | 1162 | 1.30 | 0.91 | 1.90 | 0.163 | 0.567 |
|  | seniors | 113 | 451 | 0.84 | 0.55 | 1.29 | 0.431 | 0.882 |
|  | elderly | 42 | 154 | 0.53 | 0.24 | 1.13 | 0.108 | 0.456 |
| *PHF6* | infants | 0 | 125 |  |  |  |  |  |
|  | children | 4 | 403 | 750309.9 | 4.52E-31 |  | 0.985 | 0.998 |
|  | AYA | 18 | 576 | 1.34 | 0.37 | 8.57 | 0.701 | 0.998 |
|  | adults | 30 | 1162 | 0.58 | 0.28 | 1.27 | 0.155 | 0.554 |
|  | seniors | 18 | 451 | 0.52 | 0.18 | 1.37 | 0.201 | 0.594 |
|  | elderly | 12 | 154 | 0.50 | 0.11 | 1.75 | 0.310 | 0.760 |
| *GATA2* | infants | 1 | 125 | 914792.7 | 1.5E-123 |  | 0.992 | 0.998 |
|  | children | 23 | 403 | 5859465 | 1.31E-13 |  | 0.985 | 0.998 |
|  | AYA | 49 | 576 | 1.51 | 0.63 | 4.45 | 0.401 | 0.864 |
|  | adults | 68 | 1162 | 1.47 | 0.82 | 2.84 | 0.223 | 0.621 |
|  | seniors | 15 | 451 | 0.93 | 0.32 | 2.64 | 0.895 | 0.998 |
|  | elderly | 5 | 154 | 0.38 | 0.02 | 2.66 | 0.394 | 0.862 |
| *SF3B1* | infants | 0 | 125 |  |  |  |  |  |
|  | children | 2 | 403 | 746082.8 | 5.24E-65 |  | 0.990 | 0.998 |
|  | AYA | 8 | 576 | 0.49 | 0.11 | 3.40 | 0.390 | 0.862 |
|  | adults | 33 | 1162 | 0.45 | 0.22 | 0.93 | **0.026** | 0.194 |
|  | seniors | 21 | 451 | 0.32 | 0.10 | 0.83 | **0.028** | 0.196 |
|  | elderly | 9 | 154 | 1.27 | 0.30 | 5.00 | 0.729 | 0.998 |

Abbreviations**:** adjusted (adj.), confidence interval (CI), exponential (E; number times 10^X^), mutation/mutated (mut.), odds ratio (OR), patients (pts), patients with mutations in genetic variables (pts. mut.), all patients with available information on the genetic variable (pts. w. info). The total numbers of patients per age group were as follows: infants n=126, children n=408, AYA n=589, adults n=1204, seniors n=518, and elderly n=217. Boldface indicates statistical significance (*p*<0.05).

## Supplemental Table S5. Univariable analyses of genetic alterations per age group for achievement of 2-year overall survival

| **Variable** | **Age Group** | **Pts. mut.** | **Pts. w. info.** | **HR** | **lower CI** | **upper**  **CI** | ***p*** | ***p adj.*** |
| --- | --- | --- | --- | --- | --- | --- | --- | --- |
| *NPM1* | infants | 0 | 126 |  |  |  |  |  |
|  | children | 29 | 406 | 0.43 | 0.18 | 1.05 | 0.063 | 0.245 |
|  | AYA | 121 | 588 | 0.62 | 0.44 | 0.88 | **0.007** | **0.046** |
|  | adults | 418 | 1196 | 0.71 | 0.61 | 0.84 | **0.000** | **0.001** |
|  | seniors | 131 | 506 | 0.70 | 0.56 | 0.88 | **0.002** | **0.019** |
|  | elderly | 54 | 204 | 1.11 | 0.80 | 1.55 | 0.515 | 0.845 |
| *FLT3*-ITD | infants | 1 | 126 | 1.11E-07 | 0 |  | 0.997 | 0.997 |
|  | children | 63 | 408 | 1.74 | 1.16 | 2.61 | **0.007** | **0.047** |
|  | AYA | 132 | 588 | 1.16 | 0.87 | 1.55 | 0.315 | 0.638 |
|  | adults | 299 | 1197 | 1.23 | 1.04 | 1.45 | **0.014** | 0.076 |
|  | seniors | 112 | 513 | 0.93 | 0.74 | 1.18 | 0.553 | 0.862 |
|  | elderly | 37 | 205 | 1.09 | 0.75 | 1.59 | 0.636 | 0.912 |
| *DNMT3A* | infants | 0 | 126 |  |  |  |  |  |
|  | children | 0 | 408 |  |  |  |  |  |
|  | AYA | 52 | 589 | 1.50 | 1.02 | 2.21 | **0.040** | 0.183 |
|  | adults | 367 | 1204 | 1.03 | 0.88 | 1.20 | 0.753 | 0.963 |
|  | seniors | 135 | 518 | 0.88 | 0.71 | 1.10 | 0.259 | 0.560 |
|  | elderly | 56 | 217 | 1.04 | 0.74 | 1.44 | 0.832 | 0.990 |
| t(8;21) | infants | 1 | 118 | 1.11E-07 | 0 |  | 0.997 | 0.997 |
|  | children | 86 | 397 | 0.60 | 0.37 | 0.97 | **0.039** | 0.181 |
|  | AYA | 47 | 555 | 0.43 | 0.24 | 0.80 | **0.007** | **0.046** |
|  | adults | 41 | 1130 | 0.35 | 0.20 | 0.61 | **0.000** | **0.003** |
|  | seniors | 7 | 472 | 0.53 | 0.22 | 1.28 | 0.157 | 0.421 |
|  | elderly | 2 | 191 | 1.17 | 0.29 | 4.75 | 0.823 | 0.990 |
| *CEBPA* | infants | 1 | 126 | 1.11E-07 | 0 |  | 0.997 | 0.997 |
|  | children | 37 | 408 | 0.51 | 0.24 | 1.09 | 0.082 | 0.279 |
|  | AYA | 85 | 586 | 0.59 | 0.39 | 0.88 | **0.010** | 0.060 |
|  | adults | 159 | 1186 | 0.77 | 0.62 | 0.97 | **0.023** | 0.120 |
|  | seniors | 47 | 498 | 0.67 | 0.47 | 0.95 | **0.026** | 0.133 |
|  | elderly | 25 | 193 | 0.61 | 0.38 | 0.98 | **0.039** | 0.181 |
| inv(16)/t(16;16) | infants | 18 | 119 | 1.11E-08 | 0 |  | 0.996 | 0.997 |
|  | children | 61 | 397 | 0.33 | 0.16 | 0.67 | **0.002** | **0.018** |
|  | AYA | 97 | 556 | 0.71 | 0.49 | 1.03 | 0.070 | 0.260 |
|  | adults | 74 | 1130 | 0.57 | 0.40 | 0.81 | **0.002** | **0.018** |
|  | seniors | 10 | 472 | 0.62 | 0.29 | 1.31 | 0.214 | 0.496 |
|  | elderly | 4 | 191 | 0.46 | 0.15 | 1.46 | 0.189 | 0.473 |
| -7 | infants | 1 | 119 | 5.10 | 0.69 | 37.93 | 0.111 | 0.349 |
|  | children | 13 | 397 | 2.92 | 1.48 | 5.77 | **0.002** | **0.018** |
|  | AYA | 13 | 556 | 3.13 | 1.66 | 5.92 | **0.000** | **0.005** |
|  | adults | 63 | 1130 | 2.57 | 1.94 | 3.39 | **2.99E-11** | **2.08E-09** |
|  | seniors | 28 | 472 | 1.74 | 1.16 | 2.58 | **0.007** | **0.046** |
|  | elderly | 11 | 191 | 1.50 | 0.76 | 2.94 | 0.240 | 0.522 |
| *IDH2* | infants | 0 | 126 |  |  |  |  |  |
|  | children | 13 | 408 | 0.64 | 0.20 | 2.00 | 0.442 | 0.785 |
|  | AYA | 39 | 589 | 1.08 | 0.67 | 1.74 | 0.763 | 0.963 |
|  | adults | 159 | 1204 | 0.99 | 0.80 | 1.23 | 0.963 | 0.997 |
|  | seniors | 93 | 518 | 0.63 | 0.48 | 0.81 | **0.000** | **0.005** |
|  | elderly | 42 | 217 | 0.79 | 0.55 | 1.13 | 0.198 | 0.476 |
| *TP53* | infants | 1 | 126 | 62.00 | 5.62 | 683.74 | **0.001** | **0.009** |
|  | children | 6 | 408 | 2.25 | 0.72 | 7.07 | 0.165 | 0.439 |
|  | AYA | 15 | 589 | 3.14 | 1.71 | 5.75 | **0.000** | **0.003** |
|  | adults | 82 | 1204 | 3.74 | 2.94 | 4.76 | **1.12E-26** | **3.12E-24** |
|  | seniors | 64 | 518 | 2.30 | 1.75 | 3.03 | **2.09E-09** | **8.33E-08** |
|  | elderly | 25 | 217 | 3.01 | 1.93 | 4.69 | **1.07E-06** | **3.31E-05** |
| *NRAS* | infants | 36 | 126 | 0.49 | 0.22 | 1.11 | 0.088 | 0.291 |
|  | children | 123 | 408 | 0.77 | 0.53 | 1.13 | 0.184 | 0.466 |
|  | AYA | 143 | 589 | 1.23 | 0.93 | 1.63 | 0.139 | 0.402 |
|  | adults | 191 | 1204 | 0.88 | 0.72 | 1.08 | 0.230 | 0.516 |
|  | seniors | 62 | 518 | 1.08 | 0.80 | 1.45 | 0.618 | 0.912 |
|  | elderly | 22 | 217 | 0.99 | 0.63 | 1.55 | 0.968 | 0.997 |
| *WT1* | infants | 9 | 126 | 1.68 | 0.60 | 4.74 | 0.324 | 0.647 |
|  | children | 61 | 408 | 2.05 | 1.38 | 3.05 | **0.000** | **0.005** |
|  | AYA | 88 | 589 | 1.34 | 0.97 | 1.85 | 0.076 | 0.270 |
|  | adults | 88 | 1204 | 1.10 | 0.84 | 1.45 | 0.486 | 0.815 |
|  | seniors | 23 | 518 | 1.37 | 0.87 | 2.15 | 0.170 | 0.447 |
|  | elderly | 4 | 217 | 0.47 | 0.15 | 1.47 | 0.193 | 0.476 |
| *KRAS* | infants | 24 | 126 | 1.64 | 0.80 | 3.36 | 0.179 | 0.459 |
|  | children | 38 | 408 | 2.27 | 1.42 | 3.62 | **0.001** | **0.007** |
|  | AYA | 33 | 589 | 1.72 | 1.06 | 2.78 | 0.027 | 0.133 |
|  | adults | 77 | 1204 | 1.10 | 0.82 | 1.48 | 0.532 | 0.859 |
|  | seniors | 17 | 518 | 1.44 | 0.87 | 2.37 | 0.154 | 0.417 |
|  | elderly | 4 | 217 | 0.67 | 0.25 | 1.81 | 0.429 | 0.782 |
| *ASXL1* | infants | 3 | 126 | 11.82 | 3.45 | 40.48 | **0.000** | **0.002** |
|  | children | 18 | 408 | 0.88 | 0.36 | 2.15 | 0.777 | 0.964 |
|  | AYA | 16 | 589 | 1.35 | 0.67 | 2.74 | 0.401 | 0.766 |
|  | adults | 52 | 1204 | 1.52 | 1.12 | 2.07 | **0.008** | **0.048** |
|  | seniors | 47 | 518 | 1.19 | 0.88 | 1.63 | 0.264 | 0.564 |
|  | elderly | 14 | 217 | 1.07 | 0.62 | 1.84 | 0.811 | 0.990 |
| del(5q) | infants | 2 | 119 | 1.54 | 0.21 | 11.28 | 0.668 | 0.923 |
|  | children | 3 | 397 | 1.20 | 0.17 | 8.58 | 0.857 | 0.997 |
|  | AYA | 11 | 556 | 1.43 | 0.64 | 3.23 | 0.384 | 0.738 |
|  | adults | 44 | 1130 | 3.08 | 2.22 | 4.27 | **1.7E-11** | **1.58E-09** |
|  | seniors | 44 | 472 | 1.90 | 1.37 | 2.63 | **0.000** | **0.002** |
|  | elderly | 17 | 191 | 3.10 | 1.85 | 5.18 | **0.000** | **0.000** |
| *GATA2* | infants | 1 | 126 | 1.11E-07 | 0 |  | 0.997 | 0.997 |
|  | children | 23 | 408 | 0.81 | 0.38 | 1.73 | 0.587 | 0.895 |
|  | AYA | 49 | 589 | 0.58 | 0.34 | 0.99 | **0.046** | 0.190 |
|  | adults | 68 | 1204 | 0.71 | 0.51 | 0.99 | **0.046** | 0.190 |
|  | seniors | 17 | 518 | 1.04 | 0.63 | 1.71 | 0.887 | 0.997 |
|  | elderly | 5 | 217 | 0.64 | 0.26 | 1.56 | 0.325 | 0.647 |
| *RUNX1* | infants | 0 | 126 |  |  |  |  |  |
|  | children | 6 | 408 | 1.92 | 0.61 | 6.03 | 0.265 | 0.564 |
|  | AYA | 23 | 589 | 1.26 | 0.70 | 2.25 | 0.437 | 0.782 |
|  | adults | 99 | 1204 | 1.70 | 1.34 | 2.16 | **0.000** | **0.000** |
|  | seniors | 82 | 518 | 1.19 | 0.92 | 1.54 | 0.196 | 0.476 |
|  | elderly | 40 | 217 | 0.95 | 0.66 | 1.36 | 0.766 | 0.963 |
| *U2AF1* | infants | 2 | 126 | 1.43 | 0.20 | 10.43 | 0.723 | 0.948 |
|  | children | 4 | 408 | 1.43 | 0.35 | 5.77 | 0.617 | 0.912 |
|  | AYA | 13 | 589 | 2.00 | 1.03 | 3.90 | **0.041** | 0.183 |
|  | adults | 25 | 1204 | 2.35 | 1.54 | 3.60 | **0.000** | **0.002** |
|  | seniors | 38 | 518 | 1.61 | 1.13 | 2.29 | **0.008** | **0.049** |
|  | elderly | 15 | 217 | 1.60 | 0.94 | 2.71 | 0.084 | 0.282 |
| -Y | infants | 1 | 119 | 1.11E-07 | 0 |  | 0.997438 | 0.997493 |
|  | children | 25 | 397 | 0.80 | 0.37 | 1.72 | 0.571 | 0.881 |
|  | AYA | 16 | 556 | 0.36 | 0.12 | 1.13 | 0.080 | 0.277 |
|  | adults | 30 | 1130 | 0.72 | 0.44 | 1.16 | 0.179 | 0.459 |
|  | seniors | 8 | 472 | 1.22 | 0.58 | 2.59 | 0.599 | 0.908 |
|  | elderly | 6 | 191 | 1.16 | 0.51 | 2.62 | 0.727 | 0.948 |
| *STAG2* | infants | 4 | 126 | 3.89E-08 | 0 |  | 0.997 | 0.997 |
|  | children | 8 | 408 | 1.20 | 0.38 | 3.76 | 0.758 | 0.963 |
|  | AYA | 14 | 589 | 1.01 | 0.45 | 2.27 | 0.980 | 0.997 |
|  | adults | 70 | 1204 | 0.80 | 0.57 | 1.12 | 0.197 | 0.476 |
|  | seniors | 32 | 518 | 1.04 | 0.70 | 1.53 | 0.855 | 0.997 |
|  | elderly | 25 | 217 | 0.91 | 0.56 | 1.46 | 0.685 | 0.937 |

Abbreviations: adjusted (adj.), confidence interval (CI), exponential (E; number times 10^X^), hazard ratio (HR), mutation/mutated (mut.), patients (pts), patients with mutations in genetic variables (pts. mut.), all patients with available information on the genetic variable (pts. w. info). The total numbers of patients per age group were as follows: infants n=126, children n=408, AYA n=589, adults n=1204, seniors n=518, and elderly n=217. Boldface indicates statistical significance (p<0.05).

# Supplemental Figures


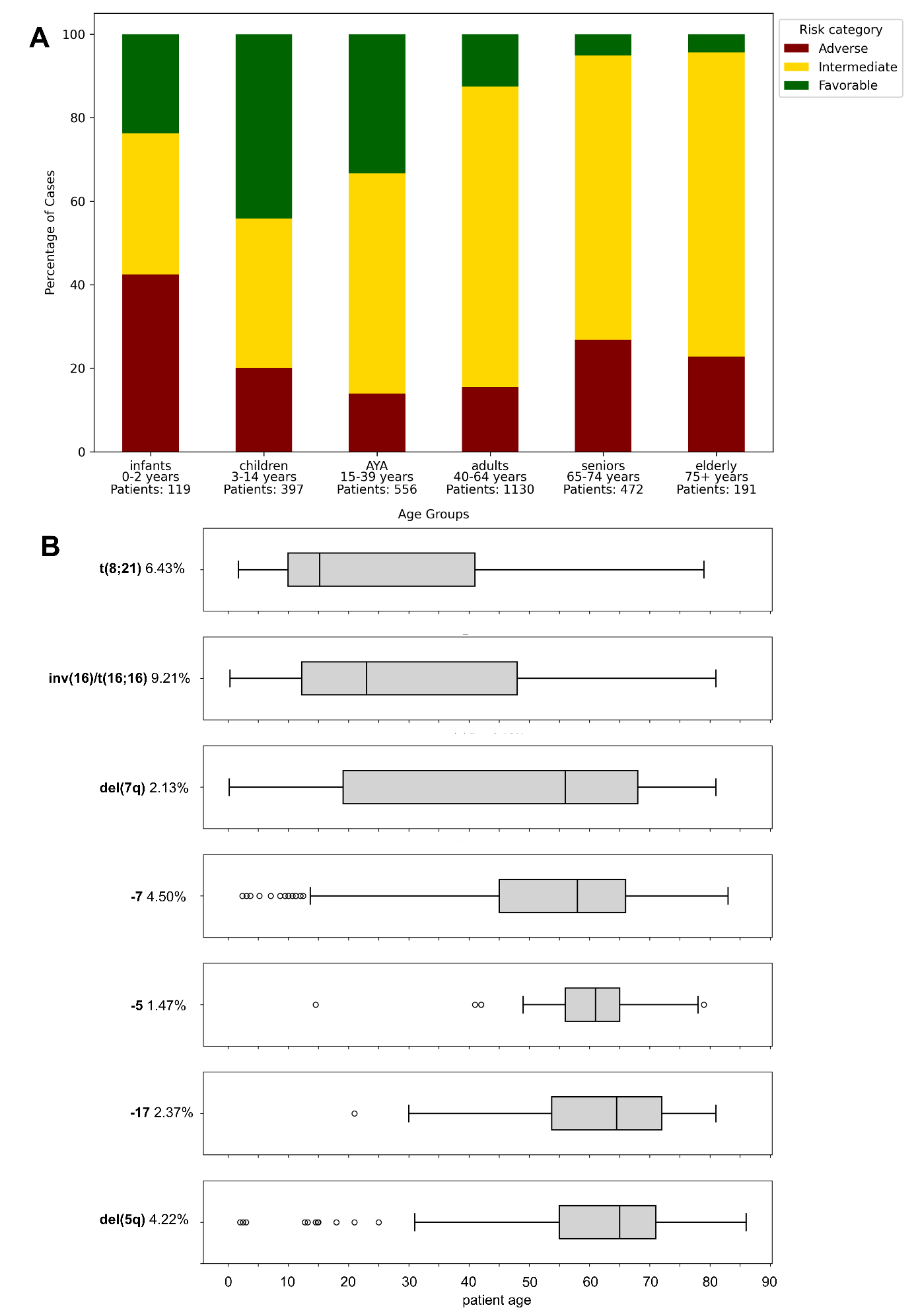


**Supplemental Figure S1. Distribution of cytogenetic alterations across age groups.** Cytogenetics are categorized according to ELN2022 recommendations and their distribution is compared between age groups (A). Favorable risk cytogenetics were more frequently found in children and decreased with age, while high risk cytogenetics were frequently found in infants, seniors, and elderly patients. Boxplots show the age distribution of the most common cytogenetic alterations (B). Boxplot: bold vertical line = median; box = interquartile range (IQR, i.e., 25th to 75th percentile); lower whisker = Q1 – 1.5 * IQR; upper whisker = Q3 + 1.5 * IQR; dots = outliers.





**Supplemental Figure S2. Model performance for prediction of complete remission (CR) and 2-year overall survival (OS).** 100 runs were performed for each model in predicting complete remission (CR) and 2-year overall survival (OS). The spread in model performance is displayed using area-under-the-curve (AUC) for the receiver-operating-characteristic (ROC; panels A and B) and Matthew’s Correlation coefficient (MCC; panels C and D). For CR prediction, Random Forest (RF) was the best performing model compared to XGBoost (XGB) and logistic regression (LR). ). For 2-year OS, XGBoost (XGB) was the best performing model compared to Random Forest (RF) and logistic regression (LR). Since LR in contrast to XGB and RF is a deterministic model resulting in an exact replication rather than varying performance per run (due to random initialization in XGB and RF models), no range of performance can be calculated for LR and LR is not shown in this plot. Boxplot: bold vertical line = median; box = interquartile range (IQR, i.e., 25th to 75th percentile); lower whisker = Q1 – 1.5 * IQR; upper whisker = Q3 + 1.5 * IQR; dots = outliers.


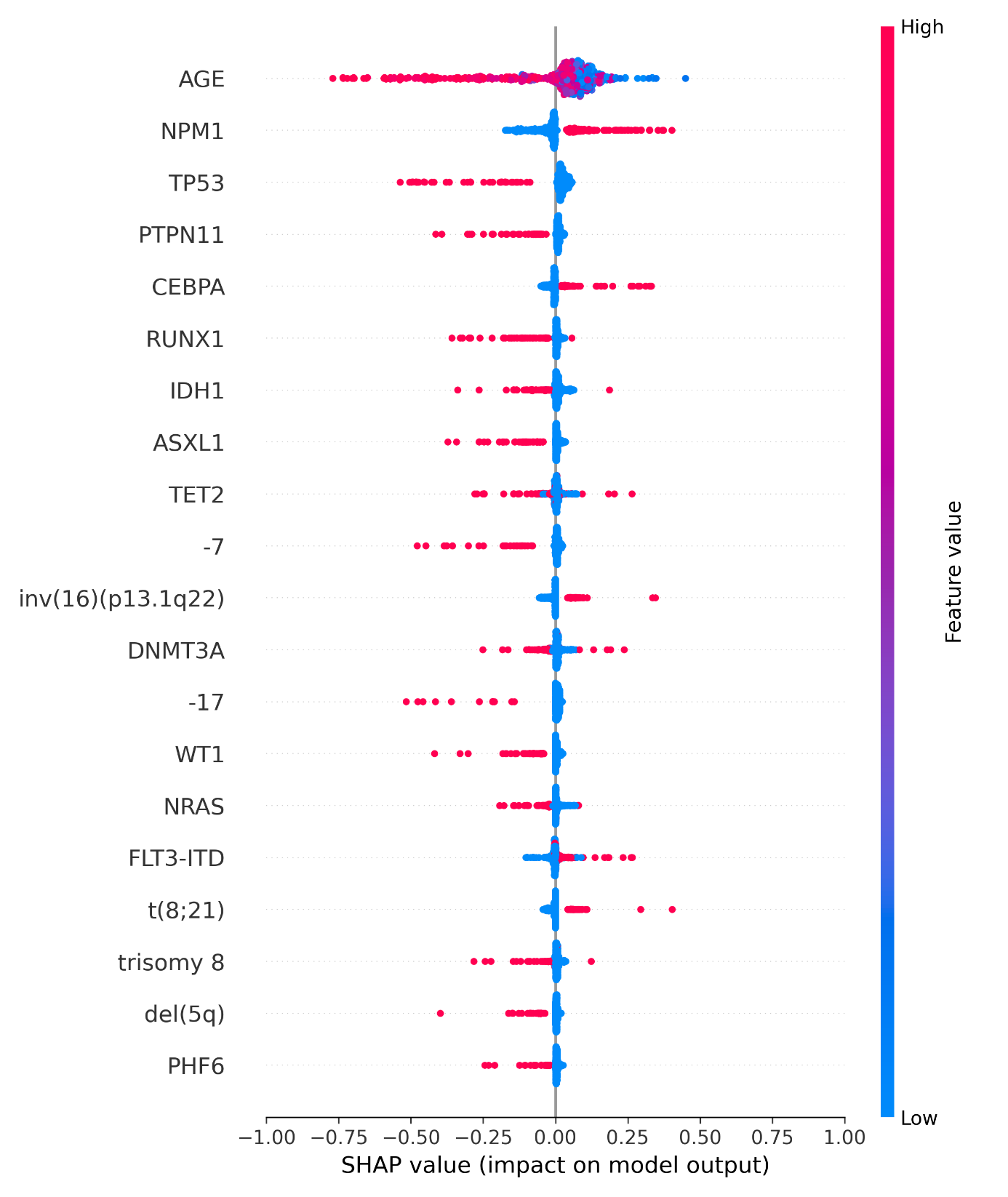
**Supplemental Figure S3. SHAP Beeswarm plot for prediction of complete remission (CR) after intensive induction therapy with XGBoost.** The influence of individual variables on model predictions are displayed for XGBoost (XGB). Automatically selected variables are listed from most influential (top) to least influential (bottom). SHAP values (x-axis) represent the impact of each variable on model decisions. Positive SHAP values (>0) indicate a favorable prediction, i.e., achievement of CR, while negative values (<0) indicate an unfavorable prediction, i.e., treatment failure. Each single dot represents a single patient. Age was treated as a continuous variable. Hence, high feature values (red) represent older patients, while lower feature values (blue) represent younger patients. Genetic alterations were treated as binary variables: present/mutated (red) vs. absent/wildtype (blue). The magnitude of the effect per patient is indicated by the SHAP value, with deviations from 0 showing either a positive (>0) or negative (<0) effect on treatment response.


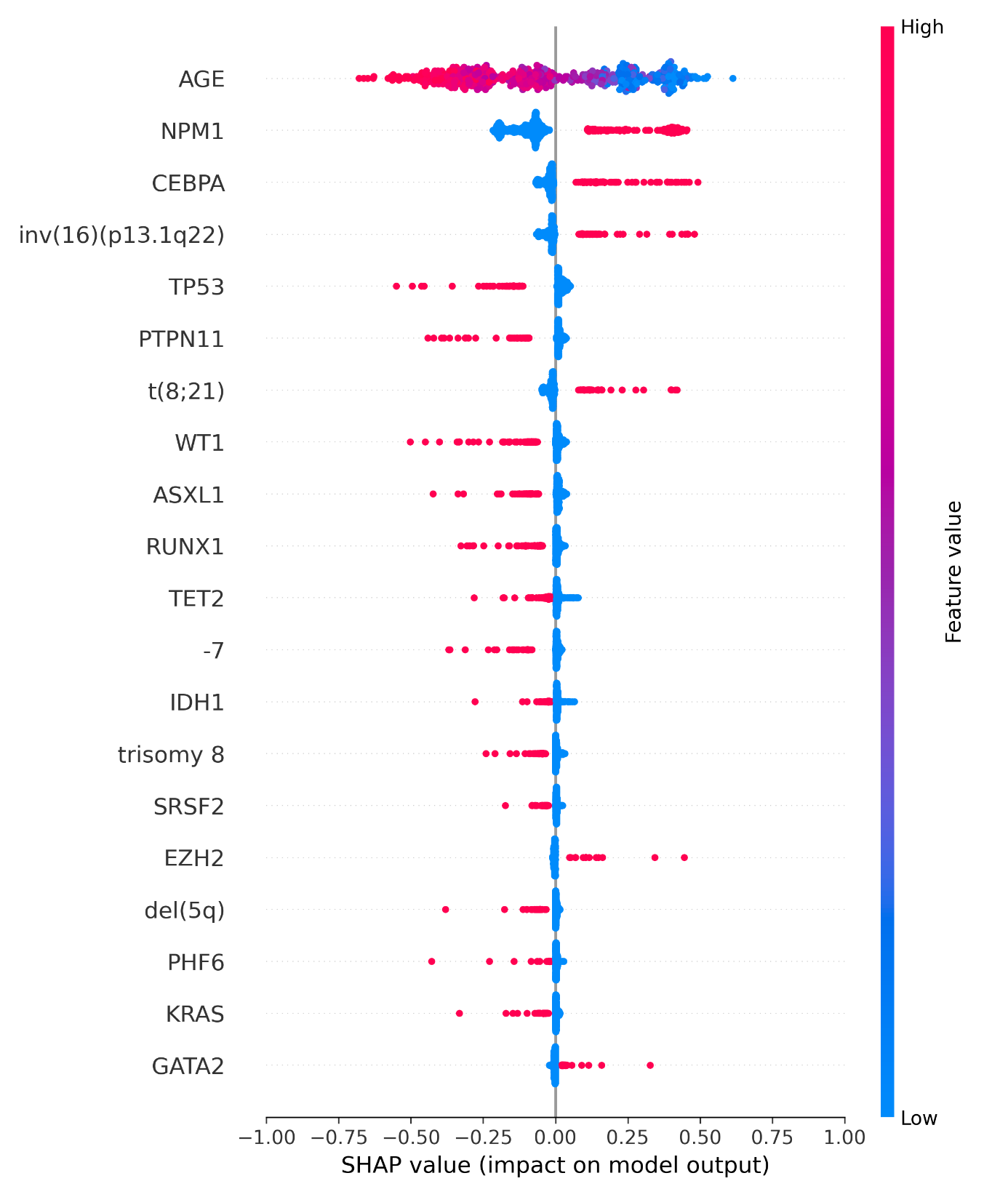
**Supplemental Figure S4. SHAP Beeswarm plot for prediction of complete remission (CR) after intensive induction therapy with Logistic Regression.** The influence of individual variables on model predictions are displayed for Logistic Regression (LR). Automatically selected variables are listed from most influential (top) to least influential (bottom). SHAP values (x-axis) represent the impact of each variable on model decisions. Positive SHAP values (>0) indicate a favorable prediction, i.e., achievement of CR, while negative values (<0) indicate an unfavorable prediction, i.e., treatment failure. Each single dot represents a single patient. Age was treated as a continuous variable. Hence, high feature values (red) represent older patients, while lower feature values (blue) represent younger patients. Genetic alterations were treated as binary variables: present/mutated (red) vs. absent/wildtype (blue). The magnitude of the effect per patient is indicated by the SHAP value, with deviations from 0 showing either a positive (>0) or negative (<0) effect on treatment response.


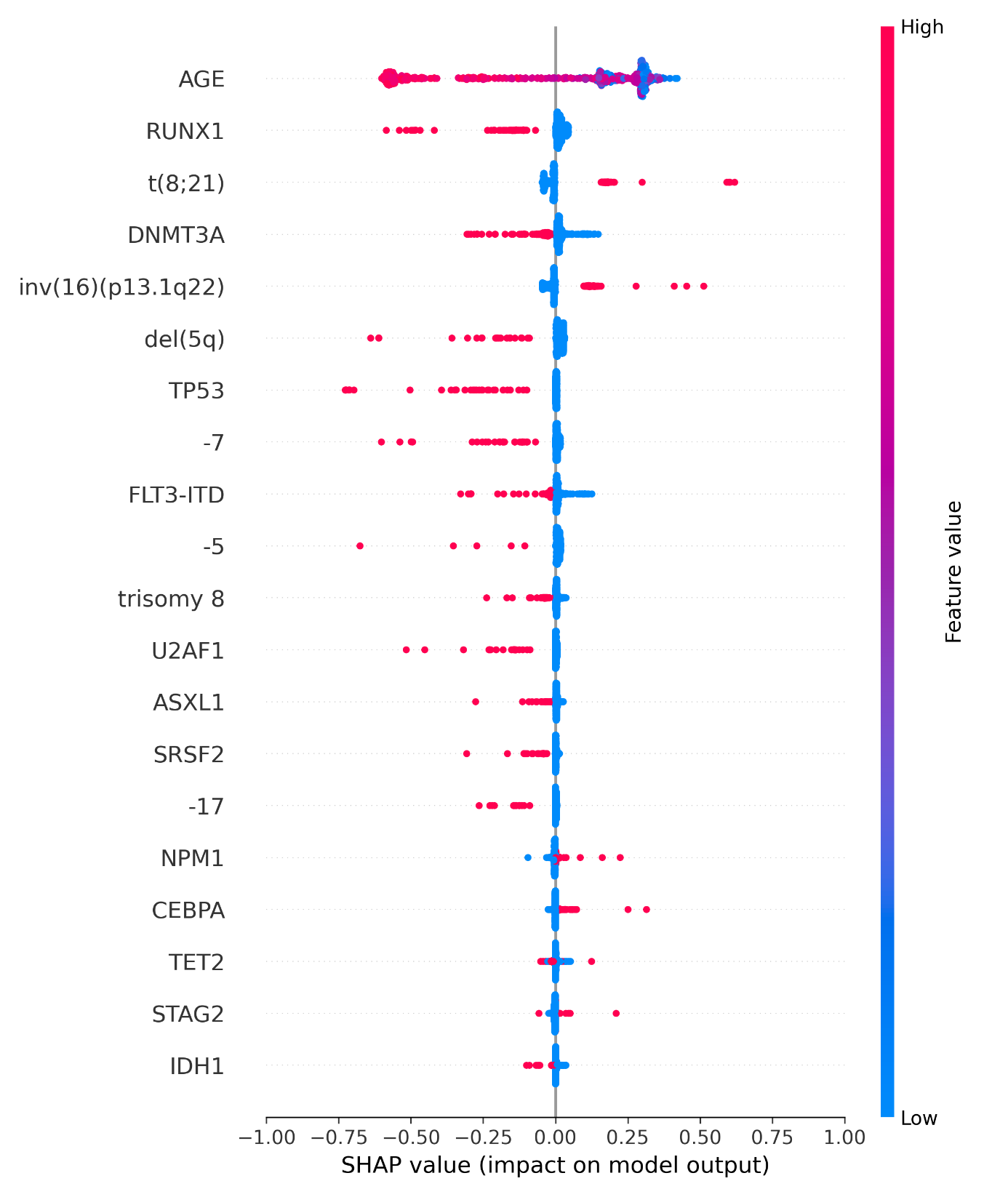
**Supplemental Figure S5. SHAP Beeswarm plot for prediction of 2-year overall survival with Random Forest.** The influence of individual variables on model predictions are displayed for Random Forest (RF). Automatically selected variables are listed from most influential (top) to least influential (bottom). SHAP values (x-axis) represent the impact of each variable on model decisions. Positive SHAP values (>0) indicate a favorable prediction, i.e., survival beyond the 2-year mark, while negative values (<0) indicate an unfavorable prediction, i.e., death within two years after diagnosis. Each single dot represents a single patient. Age was treated as a continuous variable. Hence, high feature values (red) represent older patients, while lower feature values (blue) represent younger patients. Genetic alterations were treated as binary variables: present/mutated (red) vs. absent/wildtype (blue). The magnitude of the effect per patient is indicated by the SHAP value, with deviations from 0 showing either a positive (>0) or negative (<0) effect on 2-year OS.


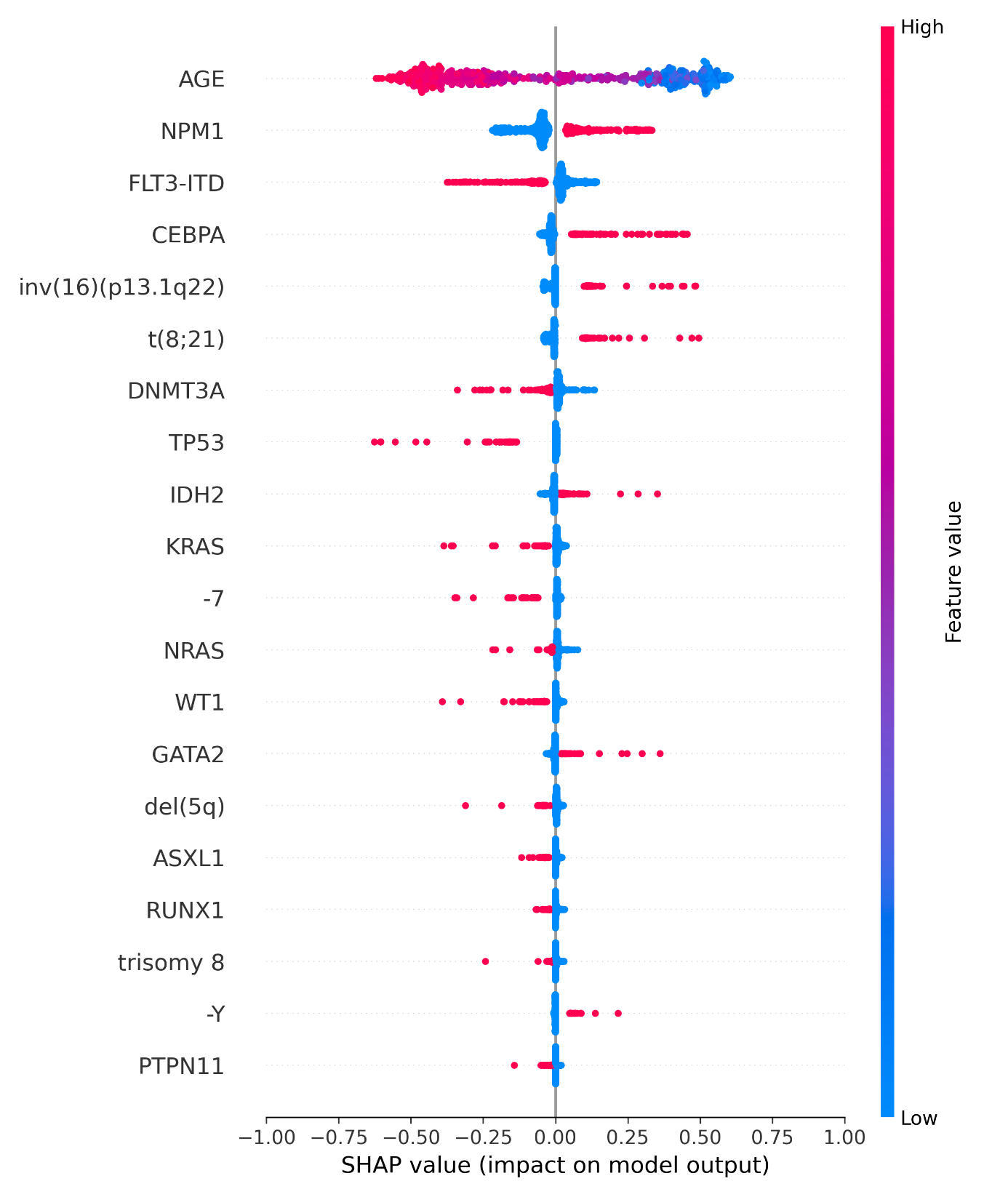


**Supplemental Figure S6. SHAP Beeswarm plot for prediction of 2-year overall survival with Logistic Regression.** The influence of individual variables on model predictions are displayed for Logistic Regression. Automatically selected variables are listed from most influential (top) to least influential (bottom). SHAP values (x-axis) represent the impact of each variable on model decisions. Positive SHAP values (>0) indicate a favorable prediction, i.e., survival beyond the 2-year mark, while negative values (<0) indicate an unfavorable prediction, i.e., death within two years after diagnosis. Each single dot represents a single patient. Age was treated as a continuous variable. Hence, high feature values (red) represent older patients, while lower feature values (blue) represent younger patients. Genetic alterations were treated as binary variables: present/mutated (red) vs. absent/wildtype (blue). The magnitude of the effect per patient is indicated by the SHAP value, with deviations from 0 showing either a positive (>0) or negative (<0) effect on 2-year OS.


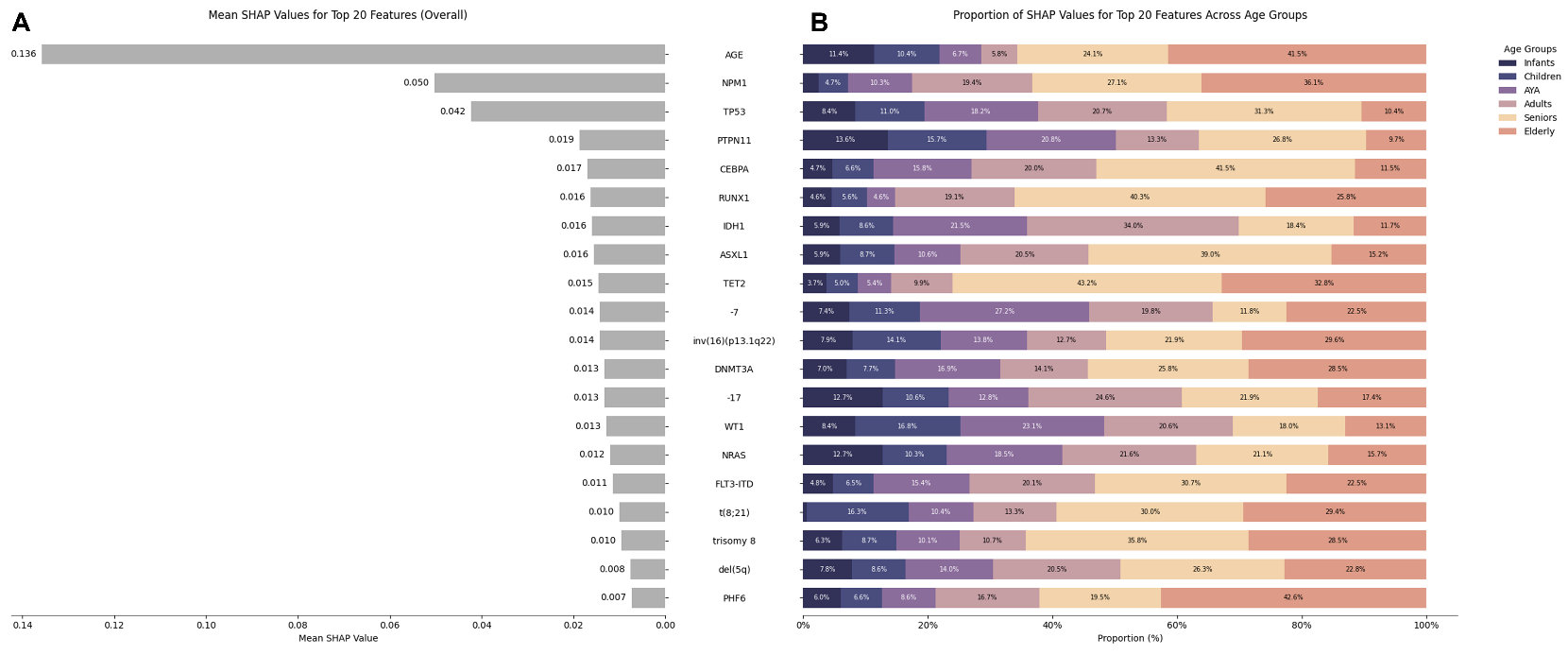


**Supplemental Figure S7. Age-group-wise impact on model inference for prediction of complete remission with XGBoost.** For XGBoost (XGB), individual variables impacting model decisions were ranked according to their mean SHAP values from highest to lowest (A). The individual contributions for a given feature were traced back to the different age groups. The effect size derived from each age group is shown as percentage of the individual contribution per age group to the variable’s mean SHAP value (B), indicating differential prognostic impacts per age group for each single variable.


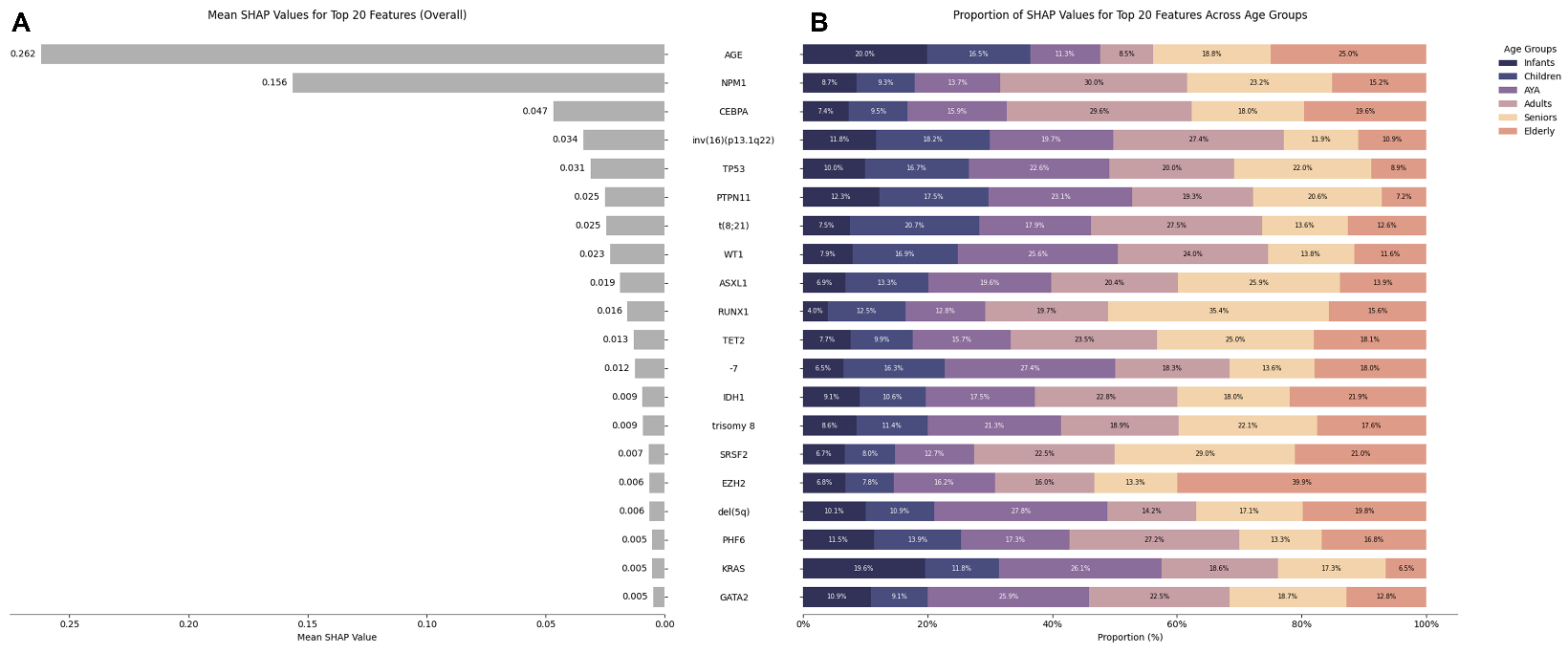


**Supplemental Figure S8. Age-group-wise impact on model inference for prediction of complete remission with Logistic Regression.** For Logistic Regression (LR), individual variables impacting model decisions were ranked according to their mean SHAP values from highest to lowest (A). The individual contributions for a given feature were traced back to the different age groups. The effect size derived from each age group is shown as percentage of the individual contribution per age group to the variable’s mean SHAP value (B), indicating differential prognostic impacts per age group for each single variable.


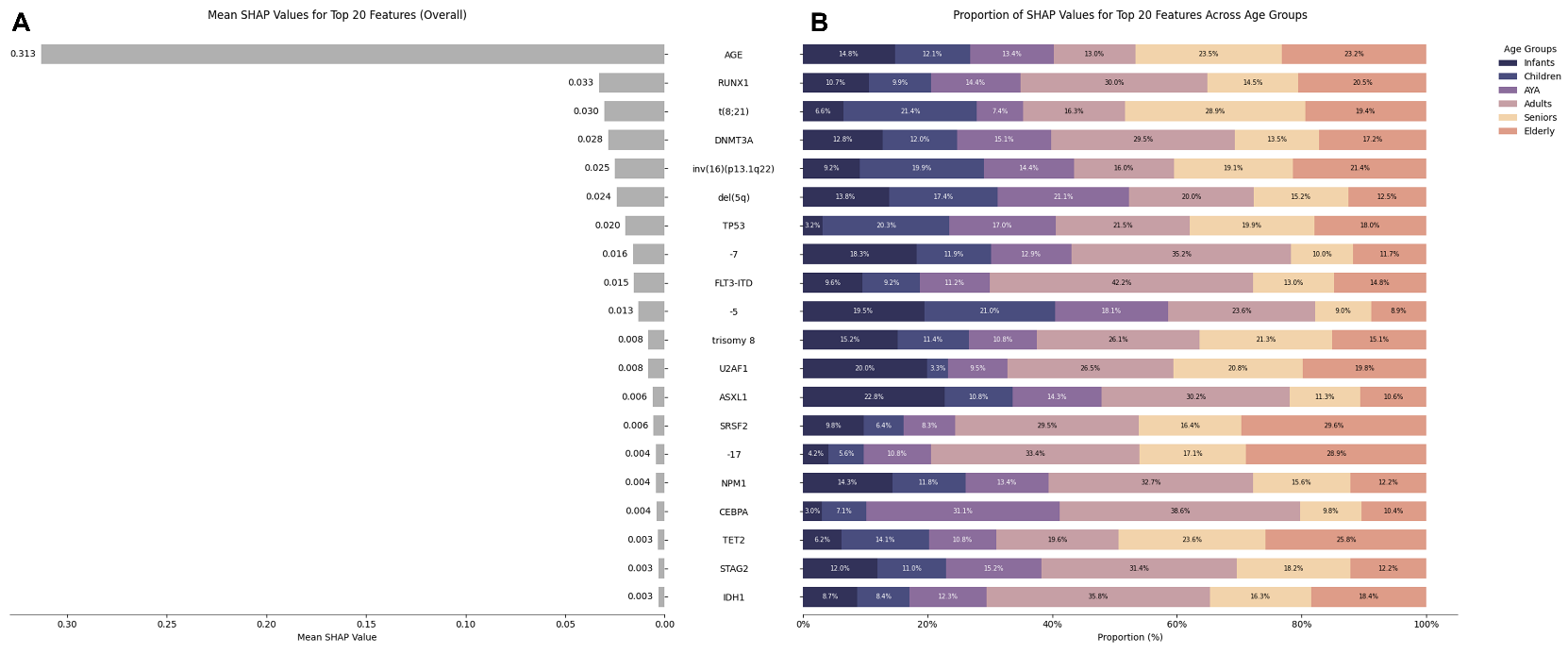


**Supplemental Figure S9. Age-group-wise impact on model inference for prediction of overall survival with Random Forest.** For Random Forest (RF), individual variables impacting model decisions were ranked according to their mean SHAP values from highest to lowest (A). The individual contributions for a given feature were traced back to the different age groups. The effect size derived from each age group is shown as percentage of the individual contribution per age group to the variable’s mean SHAP value (B), indicating differential prognostic impacts per age group for each single variable.


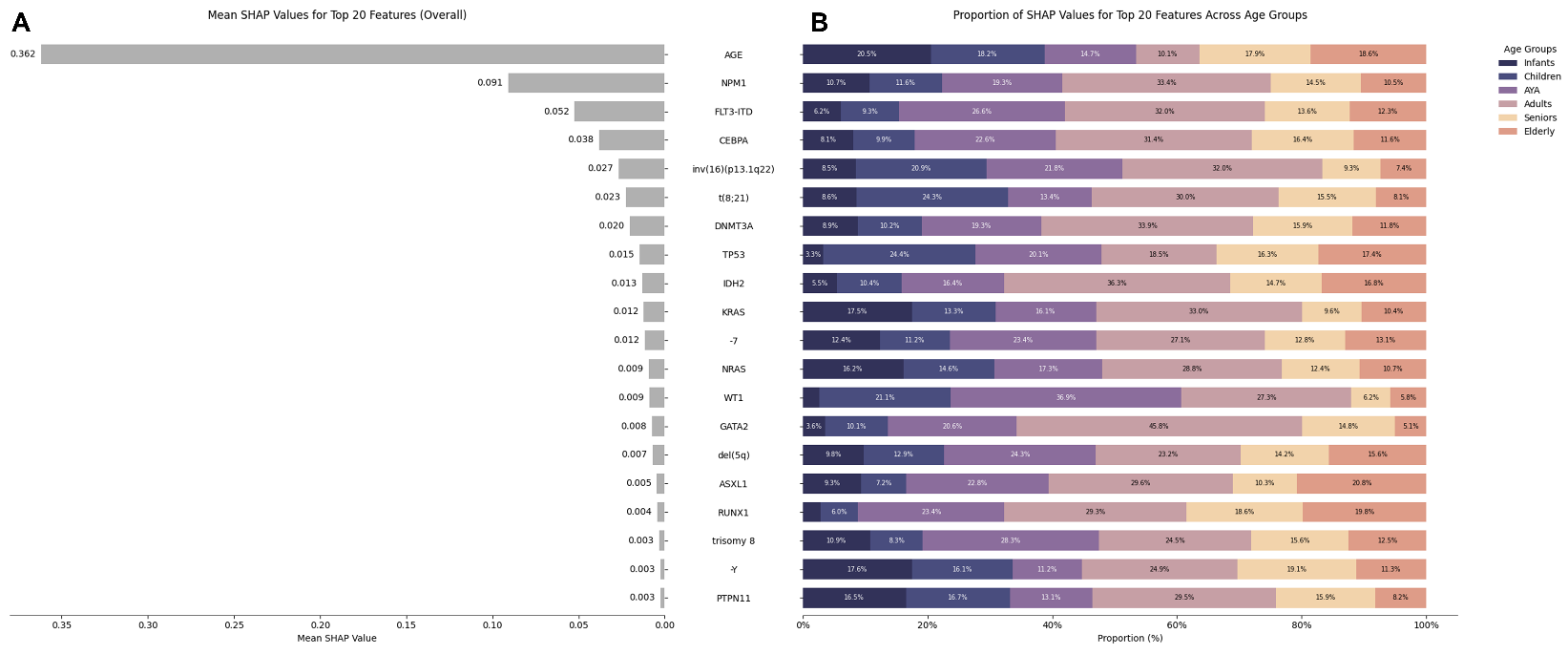


**Supplemental Figure S10. Age-group-wise impact on model inference for prediction of overall survival with Logistic Regression.** For Logistic Regression (LR), individual variables impacting model decisions were ranked according to their mean SHAP values from highest to lowest (A). The individual contributions for a given feature were traced back to the different age groups. The effect size derived from each age group is shown as percentage of the individual contribution per age group to the variable’s mean SHAP value (B), indicating differential prognostic impacts per age group for each single variable.


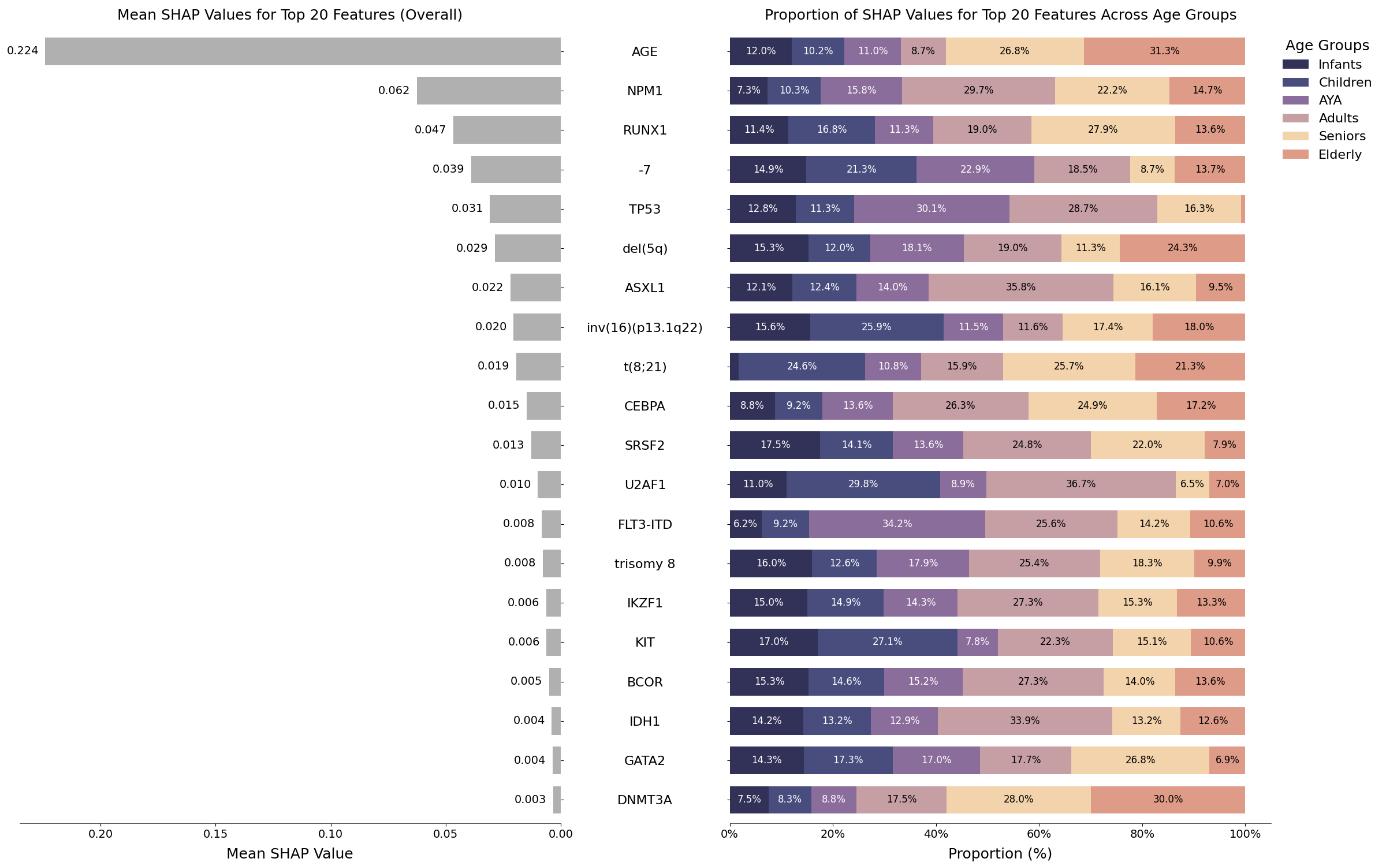


**Supplemental Figure S11. Age-group-wise impact on model inference for prediction of complete remission in female patients with Random Forest.** For Random Forest (RF), individual variables impacting model decisions were ranked according to their mean SHAP values from highest to lowest (A). The individual contributions for a given feature were traced back to the different age groups. The effect size derived from each age group is shown as percentage of the individual contribution per age group to the variable’s mean SHAP value (B), indicating differential prognostic impacts per age group for each single variable. We observed that no single variable had a uniform contribution to the overall SHAP values across all age groups, but rather that contributions to model explainability varied largely between age groups per variable.


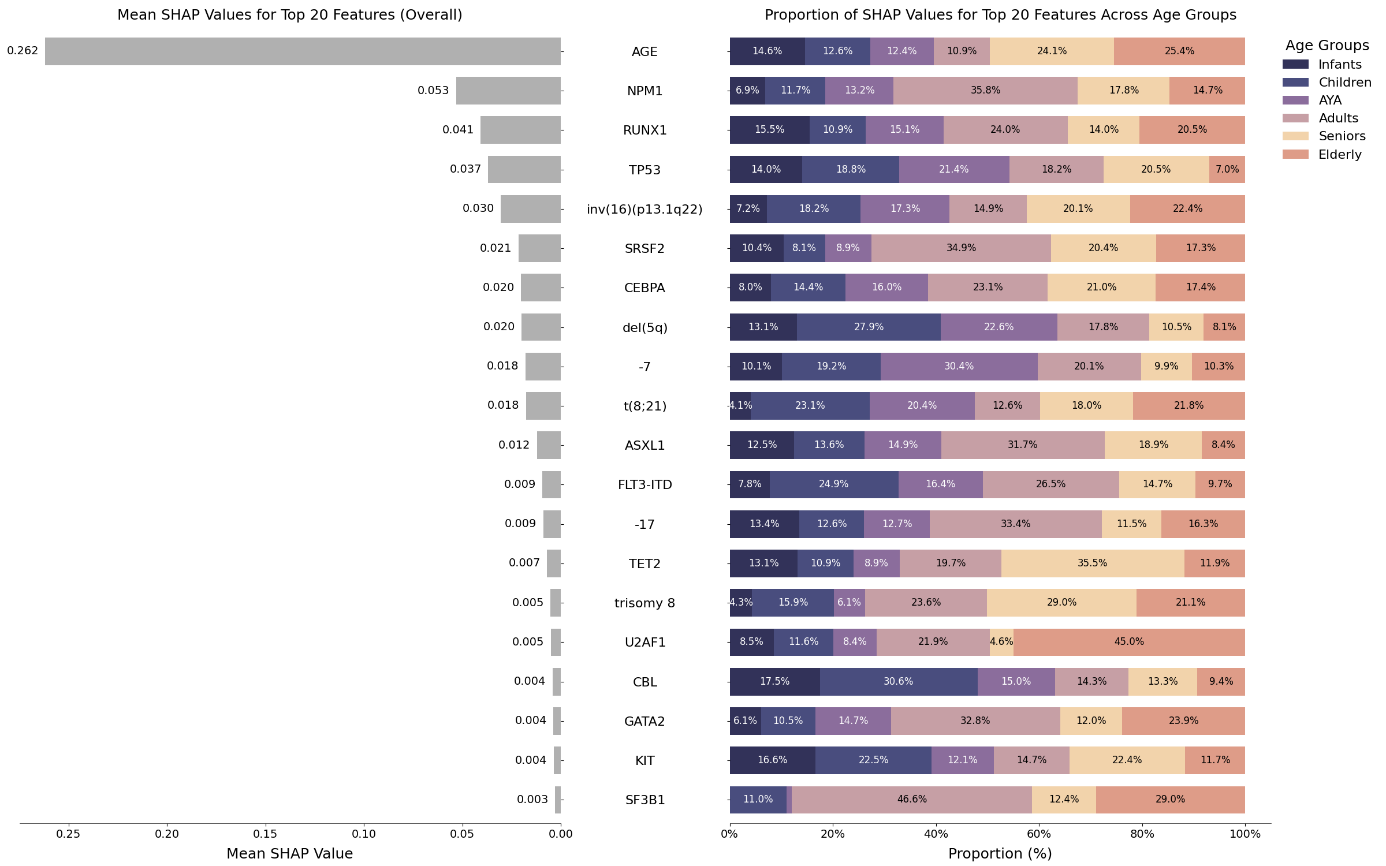


**Supplemental Figure S12. Age-group-wise impact on model inference for prediction of complete remission in male patients with Random Forest.** For Random Forest (RF), individual variables impacting model decisions were ranked according to their mean SHAP values from highest to lowest (A). The individual contributions for a given feature were traced back to the different age groups. The effect size derived from each age group is shown as percentage of the individual contribution per age group to the variable’s mean SHAP value (B), indicating differential prognostic impacts per age group for each single variable. We observed that no single variable had a uniform contribution to the overall SHAP values across all age groups, but rather that contributions to model explainability varied largely between age groups per variable.


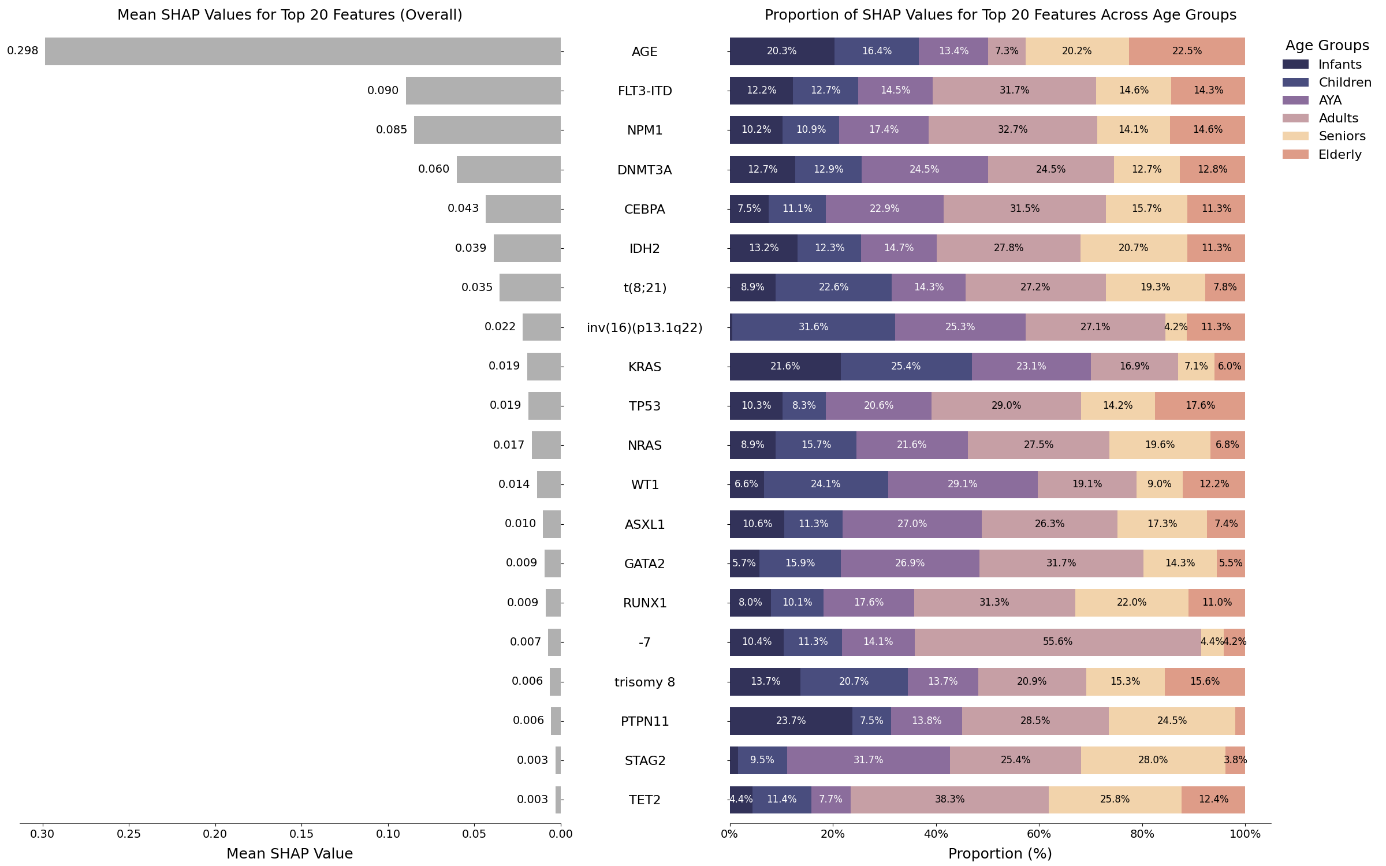


**Supplemental Figure S13. Age-group-wise impact on model inference for prediction of 2-year overall survival in female patients with XGBoost.** For XGBoost (XGB), individual variables impacting model decisions were ranked according to their mean SHAP values from highest to lowest (A). The individual contributions for a given feature were traced back to the different age groups. The effect size derived from each age group is shown as percentage of the individual contribution per age group to the variable’s mean SHAP value (B), indicating differential prognostic impacts per age group for each single variable.


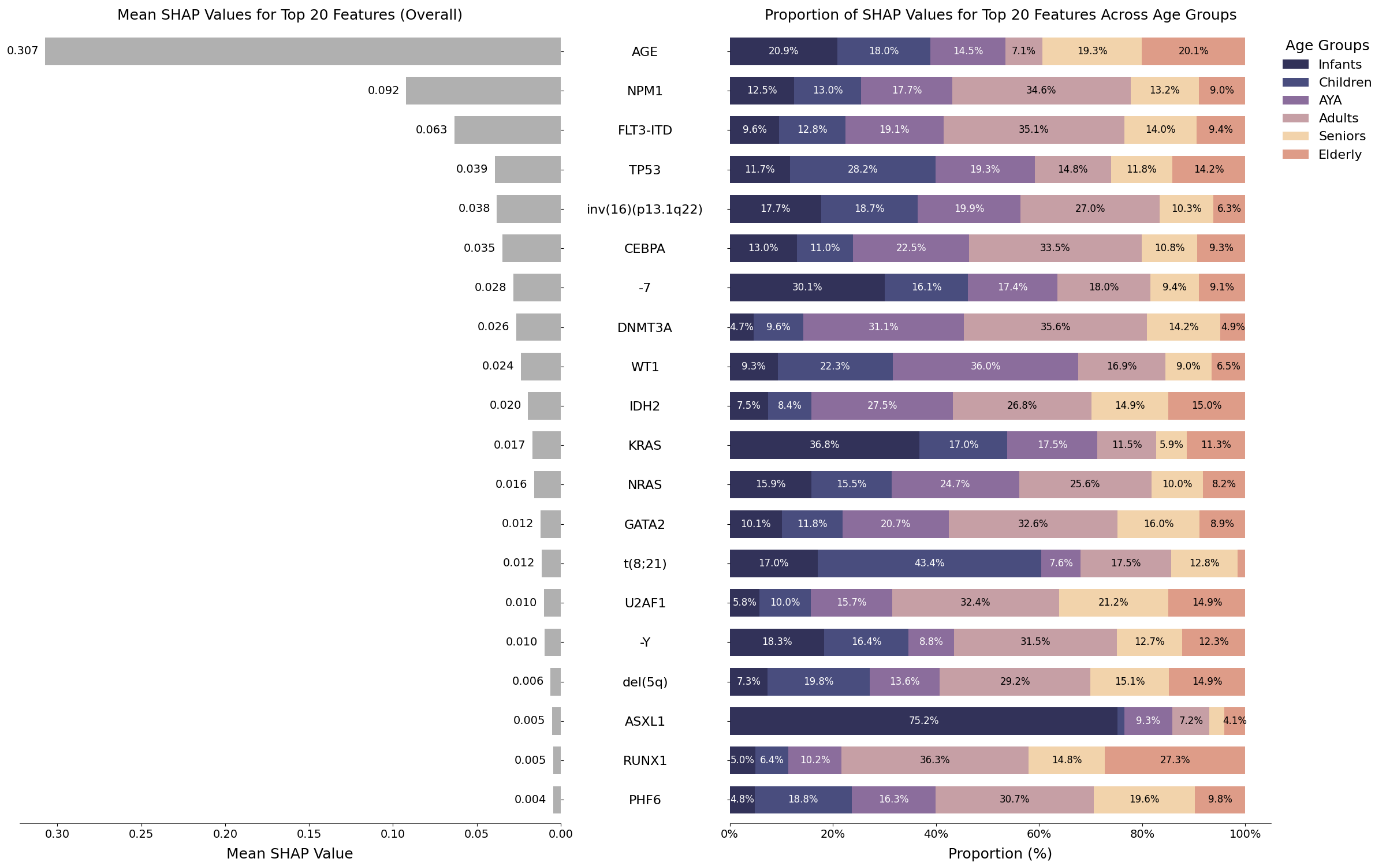


**Supplemental Figure S14. Age-group-wise impact on model inference for prediction of 2-year overall survival in male patients with XGBoost.** For XGBoost (XGB), individual variables impacting model decisions were ranked according to their mean SHAP values from highest to lowest (A). The individual contributions for a given feature were traced back to the different age groups. The effect size derived from each age group is shown as percentage of the individual contribution per age group to the variable’s mean SHAP value (B), indicating differential prognostic impacts per age group for each single variable.
